# Charge Dynamics as Probed by Small Signal-Modulated Photocurrent and Impedance Spectroscopy of Metal Vanadate Semiconductors and Alloys

**DOI:** 10.1021/acsaom.5c00154

**Published:** 2025-07-25

**Authors:** Juan Carlos Expósito-Gálvez, Abhishek Rawat, Krishnan Rajeshwar, Gerko Oskam

**Affiliations:** † Center for Nanoscience and Sustainable Technologies (CNATS). Department of Physical, Chemical and Natural Systems, 16772 Universidad Pablo de Olavide, Sevilla 41013, Spain; ‡ Department of Chemistry & Biochemistry, 12329 The University of Texas at Arlington, Arlington, Texas 76019, United States

**Keywords:** photoelectrochemistry, charge transfer, carrier recombination, external quantum efficiency, n-type semiconductors

## Abstract

This study presents the combined and complementary use of two techniques, namely, intensity modulated photocurrent spectroscopy (IMPS) and photoelectrochemical impedance spectroscopy (PEIS) for the study of partially or wholly replacing copper with an alkaline earth metal (Mg, Ca) in a metavanadate compound matrix (MV_2_O_6_). To this end, the three reference compounds (CuV_2_O_6_, CaV_2_O_6_, MgV_2_O) and two alloy compositions (Mg_0.1_Cu_0.9_V_2_O_6_ and Ca_0.1_Cu_0.9_V_2_O_6_), obtained from a solution combustion technique, were screen-printed on fluorine-doped tin oxide glass (FTO) substrates. Wavelength discrimination for optical excitation of these semiconductor samples was provided by using high-power LED (front-side) illumination with different wavelengths, UV (λ = 370 nm), blue (λ = 455 nm), green (λ = 535 nm), and red (λ = 670 nm). A nonunity photogeneration yield of mobile charge carriers was seen in a trend reminiscent of previous data on binary oxides or even for other types of ternary copper oxides from other laboratories. The dynamic aspects of charge transfer and carrier recombination are discussed using the combined IMPS-PEIS data along with other optical (e.g., diffuse reflectance spectroscopy) and photoelectrochemical data.

## Graphical abstract

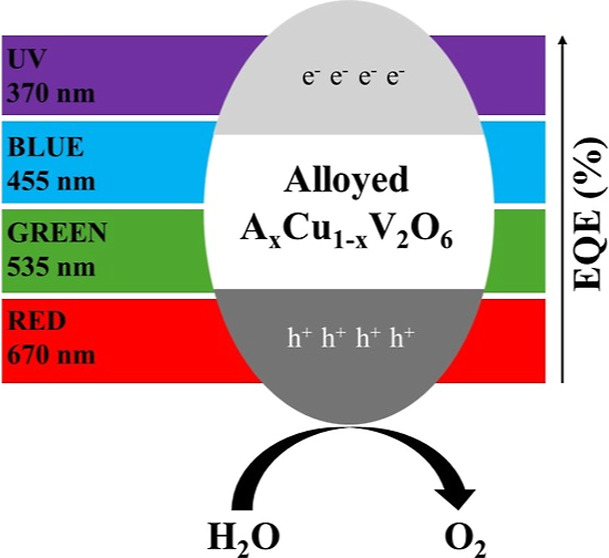

## 1 Introduction

Tuning the chemical composition or alloying metallic elements within a compound semiconductor framework is a powerful method in a materials chemist’s toolbox to tweak its properties for a targeted application. Cases in point are the semiconductor alloys in the group III–V (13–15) family where the energy band gaps are tuned for optoelectronic device (e.g., diode lasers, displays, solar cells) use by alloying the cationic component. However, other than perhaps ternary oxide perovskites, examples of chemical modification or alloying of ternary oxide semiconductors are only emerging of late.^1–13^ In this study, we explore the dynamic consequences (see below) of partially or wholly replacing copper with an alkaline earth metal (Mg, Ca) in a metavanadate compound matrix (MV_2_O_6_). This is a companion study that builds on our previous efforts on both *ortho*- and *pyro*-vanadates (M_2_V_2_O_7_).^14–16^ Closely related studies in other laboratories on a variety of ternary copper vanadates^2,5,8,9,11,12^ are also noted here. This alloying strategy was motivated by the potential of alkaline earth metals, such as Mg and Ca, to enhance water adsorption properties and alter the electronic structure in a way that could reduce recombination losses, thereby improving photocarrier generation and injection efficiency.^14–16^

Time-dependent perturbation is very effective for studying the dynamic behavior of semiconductor–electrolyte interfaces. The two most common types of perturbation are illumination with modulated intensity at a fixed electrode potential or modulation of the electrode potential at constant illumination. The corresponding techniques, namely intensity modulated photocurrent spectroscopy (IMPS) and photoelectrochemical impedance spectroscopy (PEIS), have been thoroughly reviewed in a monograph.^17^ In what follows, we use (P)­EIS for the cases when the EIS technique was used with or without illumination of the semiconductor surface (i.e., in the dark). The complementarity of IMPS and PEIS for gleaning insights into dynamic processes such as carrier recombination or interfacial charge transfer is well exemplified by the results reported by other authors on binary compound semiconductors such as GaAs, CdS, and InP,^18,19^ as well as metal oxides.^20,21^

One of us has previously used IMPS for the study of a ternary oxide semiconductor, namely, CuBi_2_O_4_.^22^ Interfacial hole transfer and carrier recombination were also studied as a function of potential for β-Cu_2_V_2_O_7_/electrolyte interfaces in a previous collaboration.^23^ In the present study, we specifically show that the recently discussed reinterpretation of declining photogeneration efficiency with wavelength in terms of being rooted in nonmobile charge carriers (rather than carrier recombination)^24^ applies to the metavanadates under discussion as well. In this context, potential issues with localized (e.g., d–d) light absorption resulting in subunity quantum yields, may be more widely prevalent than previously envisioned.

## 2 Experimental Section

### 2.1 Photoanode Fabrication

The metal vanadates CuV_2_O_6_, CaV_2_O_6_, MgV_2_O_6_, and the alloy compositions Mg_0.1_Cu_0.9_V_2_O_6_ and Ca_0.1_Cu_0.9_V_2_O_6_ were prepared in polycrystalline powder form by solution combustion synthesis (SCS) according to previously reported work,^14,15,25^ where detailed characterization and elemental analysis confirmed their exact composition. In this work, we mainly focus on the electrochemical performance of these materials.

The photoelectrodes were fabricated using a semiautomatic screen-printing system (ATMA AT-45PA). The printing paste was formulated using 0.1 g of the SCS-derived powder, which was finely ground in an agate mortar to ensure particle size homogeneity and then dispersed in 10 mL isopropanol in an ultrasonic bath for 20 min. Subsequently, 1 mL terpineol (86480, Sigma-Aldrich) was added and the dispersion was magnetically stirred for 20 min. Separately, 0.03 g ethyl cellulose 100 cP (247499, Sigma-Aldrich) was dissolved in 10 mL isopropanol and magnetically stirred at 90 °C for 1 h. Finally, the powder dispersion and the ethyl cellulose solution were combined, magnetically stirred, and sonicated for 20 min. The excess solvent was removed in a rotatory evaporator until the paste reached the desired density and viscosity.^26,27^ The procedure was independently repeated for each material.

Screen printing was performed employing a screen with a mesh opening of 68 μm, nominal thread diameter of 40 μm, open area of 37.6%, mesh thickness of 65 μm, theoretical ink volume of 24.4 cm^3^/m^2^ and deposition area of 1 cm^2^. Prior to deposition, the FTO substrates (Xop Glass, FTO TEC 15, 12–15 Ω/square) were cleaned in an ultrasonic bath for 20 min in each of the following solvents, in the given order: Milli-Q water with Hellmanex III (Z805939 Sigma-Aldrich), Milli-Q water, ethanol, isopropanol, and acetone. Finally, the substrates were dried under a nitrogen stream. To obtain the optimal film thickness of about 6 μm (see below), as defined by reaching saturation of the photocurrent upon increasing the thickness, 5 layers were subsequently printed; between deposition steps, the films were heated on a hot plate at 125 °C for 10 min. The films were finally heated to 550 °C using a linear 40 min ramp and sintered at that temperature for 1 h. The samples were left to cool down to room temperature before use.

### 2.2 Structural, Morphological, and Optical Characterization

Powder X-ray diffraction (PXRD) was performed on a Bruker D8 Discover diffractometer operating at 50 kV and 1 mA for Cu Kα (λ = 0.15418 nm), in the range of 12.5° to 60° with an increment step of 0.01° per 0.05 s. The surface morphology and film thickness were examined using a Zeiss Gemini 300 field emission scanning electron microscopy (FE-SEM) in both top-view and cross-section modes.

Diffuse reflectance spectroscopy (DRS) was performed using a UV/vis/NIR spectrophotometer model FSL1000-DD-STM Edinburgh Instruments equipped with an integrating sphere and an Xe lamp as illumination source in the range of 300 to 800 nm, with a dwell time of 1 s and step size of 1 nm. The total reflectance (*R*) spectra were calculated as *R*(λ) = *R*_sample_(λ)/*R*_reference_(λ), where *R*_reference_(λ) corresponds to the FTO reflectance. As the integrating sphere collects both diffuse and specular components, the total reflectance was measured. With the FTO substrate as reference, all light that is not absorbed is (eventually) collected, and the absorptance (*A*) was calculated directly as *A*(λ) = 1 – *R*(λ). Under these conditions, the absorptance can be considered equivalent to the light-harvesting efficiency ( *LHE* ) of the films (i.e., *LHE* (λ) ≈ *A*(λ)), representing the fraction of incident photons absorbed at each wavelength. The absorbance (Abs) was determined using Abs­(λ) = −ln­(1 – *A*(λ)). The absorption coefficient (α) was obtained through α­(λ) = Abs­(λ)/*d*, where *d* is the thickness of the photoelectrode (about 6 μm). Finally, the light penetration depth (δ_p_) was determined using δ_p_(λ) = 1/α­(λ).

The emission spectra of the light-emitting diodes (LEDs) were obtained through an optical fiber using an Ocean Optics (USB 4000) spectrometer. Attenuated total reflectance-Fourier transform infrared spectroscopy (ATR–FTIR) was performed on a JASCO FTIR-4700 instrument in the 4000–400 cm^–1^ wavenumber range.

### 2.3 Photoelectrochemical (PEC) Measurements and Photocurrent/Electrochemical Impedance Spectroscopy

The PEC measurements were performed using a three-electrode cell configuration and front-side illumination (i.e., from the electrolyte solution side) in a single-compartment electrochemical cell equipped with a quartz window, using a Pt wire as counter electrode and Ag/AgCl (3 M KCl) as reference. The electrolyte used was 0.1 M phosphate buffer at pH 6.5. The measurements were performed using an Autolab system (PGSTAT302N) and a solar simulator (ABET 1100) with an AM 1.5G filter as the light source, which was calibrated at 100 mW/cm^2^ using a Si photodiode (Newport model 91150), at a scan rate of 20 mV/s. The applied potential was converted to the potential versus the reversible hydrogen electrode (RHE) through *E* (vs RHE) = *E* (vs Ag/Ag/Cl) + *E*_Ag/Ag/Cl_ (reference) + 0.0591 pH, where *E*_Ag/Ag/Cl_ (reference) = 0.21 V vs NHE (normal hydrogen electrode). The submerged area in contact with the electrolyte was 0.785 cm^2^.

The IMPS measurements were carried out in the frequency range from 10^5^ Hz to 10^–2^ Hz under high-power LED illumination using different wavelengths: UV (λ = 370 nm), blue (λ = 455 nm), green (λ = 535 nm), and red (λ = 670 nm). Front-side illumination was employed with the LED photon source was calibrated with a Si-photodiode (Hamamatsu S12698-2) at the same photon flux of 1.6 × 10^16^ cm^–2^ s^–1^ at different applied potentials, and at different photon fluxes at a fixed applied potential of 1.8 V vs RHE. The modulation amplitude was about 10% of the base light intensity, and the response linearity was tested and confirmed through Lissajous plots. Before each IMPS measurement, a 15 min chronoamperometry measurement was performed to establish a steady-state condition and confirm the response stability. Normalization was performed by determining the number density of incident photons to obtain real values of the external quantum efficiency ( *EQE* ).

IMPS results are typically interpreted through the transfer function, *H*(*f*), which is defined as the ratio of modulated photocurrent to the modulated light intensity and represented as a complex number: *H*(*f*) = Re­(*H*) + i Im­(*H*). Here, *f* is the frequency of the small-amplitude sinusoidal modulation that is superimposed on a constant bias light intensity. When an LED with a specific wavelength is used, the modulated light intensity can be described in terms of photon flux (cm^–2^ s^–1^), while the modulated photocurrent is converted to electron flux (cm^–2^ s^–1^). As a result, *H*(*f*) represents the complex, small-signal, frequency-dependent *EQE* .^26–37^

Electrochemical impedance spectroscopy (EIS) was performed with a potential perturbation amplitude of 10 mV (p/p) in the range from 10^5^ Hz to 10^–2^ Hz under dark and high-power LED blue (λ = 455 nm) illumination at a photon flux of 1.6 × 10^16^ cm^–2^ s^–1^ as a function of the applied potential. The latter technique variant is referred to as photoelectrochemical EIS or PEIS in what follows. The effective capacitance (*C*_eff_) was calculated from the constant phase element (CPE) parameters extracted from the equivalent circuit (see below) using the expression: *C*_eff_ = (*Q*·*R* ^1–*n*^)^1/*n*^, where *Q* is the CPE value (with units of *s* ^*n*^/Ω) and *n* is the exponent indicating the deviation from ideal capacitive behavior, and *R* (with units of Ω) is the resistance in parallel with the CPE. It should be noted that the estimation of *C*_eff_ becomes less reliable for CPE exponents below about 0.90, where the system significantly deviates from ideal capacitive behavior.^38,39^

## 3 Results and Discussion

### 3.1 Structural, Morphological, and Optical Attributes

Photographs of the 5-layer screen-printed electrodes are presented in Figure S1 in the Supporting Information. The films present good homogeneity, with different colors for CaV_2_O_6_ and MgV_2_O_6_, and the same color with different hues for CuV_2_O_6_ and the alloy modifications, Mg_0.1_Cu_0.9_V_2_O_6_ and Ca_0.1_Cu_0.9_V_2_O_6_. Figure S2 shows the X-ray diffraction patterns for each vanadate compound deposited on FTO. The CaV_2_O_6_ and MgV_2_O_6_ films presented a monoclinic structure (PDF #73-0186 and PDF #71-1651, respectively), while the CuV_2_O_6_ film and the alloy modifications Mg_0.1_Cu_0.9_V_2_O_6_ and Ca_0.1_Cu_0.9_V_2_O_6_ adopted a triclinic structure (PDF #75-1054). The last two samples had grains with preferred orientation as diagnosed by the peak located at 29.3° in the (201) plane. The peaks at 26.5°, 33.8°, 37.9° and 51.7° corresponded to the FTO substrate. The film pattern data agree very well with those of the corresponding SCS-derived powder samples. In previous work, elemental analysis using XPS confirmed the stoichiometry of the compounds, and detailed Rietveld analysis was performed on the XRD patterns of the powder materials, confirming the phase purity of the materials.^15^

SEM images of the photoelectrodes are presented in Figure S3 (top-view) and Figure S4 (cross-section) revealing that the morphology and particle shape of CuV_2_O_6_, Mg_0.1_Cu_0.9_V_2_O_6_ and Ca_0.1_Cu_0.9_V_2_O_6_ films were similar, with a particle size of about 2 μm and a homogeneous distribution. In contrast, the MgV_2_O_6_ film exhibited larger and more agglomerated particles, while the CaV_2_O_6_ film showed the smallest particle size of about 0.4 μm. The film thickness was approximately 6 μm for all samples, obtained by screen-printing 5 layers. The films are generally porous, which implies that in the electrochemical experiments the FTO and the metal oxide semiconductor are both in contact with the electrolyte solution.

Figure 1a shows the DRS data for the five samples under study. Figure 1b illustrates the corresponding light-harvesting efficiency ( *LHE* ) spectra derived from these measurements (see Section 2.2). The higher *LHE* values were obtained for CuV_2_O_6_, with 74% for UV (λ = 370 nm), 83% for blue (λ = 455 nm), and 82% for green (λ = 535 nm) illumination. The Mg_0.1_Cu_0.9_V_2_O_6_ and Ca_0.1_Cu_0.9_V_2_O_6_ alloy samples exhibited a decrease of about 5% in *LHE* relative to CuV_2_O_6_, while CaV_2_O_6_ and MgV_2_O_6_ absorb significantly less. Figure 1c presents the light penetration depth for the samples of interest compared to the three high-power LED spectra of different wavelengths used in the PEC measurement.

**Figure 1:**
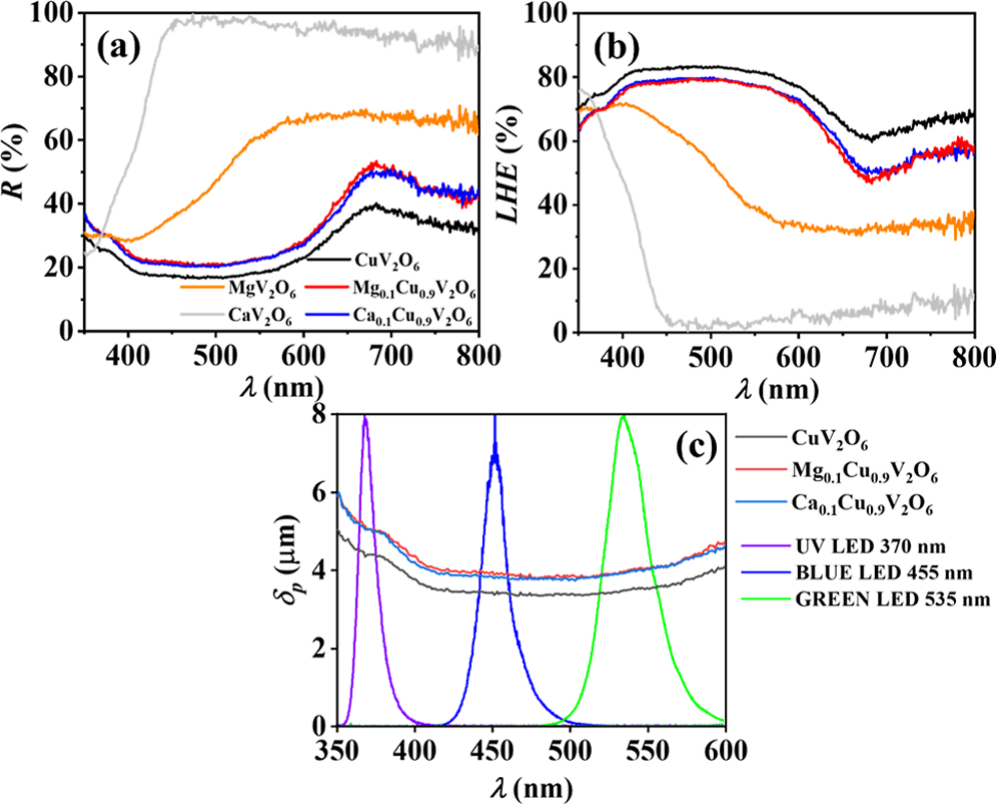
(a) Diffuse reflectance spectroscopy (*R*) data (b) light harvesting efficiency (*LHE*) and (δ_p_) (c) light penetration depth for CuV_2_O_6_, CaV_2_O_6_, MgV_2_O_6_, Ca_0.1_Cu_0.9_V_2_O_6_ and Mg_0.1_Cu_0.9_V_2_O_6_ films screen-printed on FTO.

The penetration depth is about 4 μm for all materials and increases somewhat in the UV and toward the NIR. Comparing to the film thickness of 6 μm, this implies that a small fraction of light is not absorbed, as also observed in the *LHE* graph. Note that the inhomogeneous morphology and the partial coverage of the FTO substrate complicates the *LHE* analysis, leading to a relatively large uncertainty in the parameter values extracted. UV light exhibited a somewhat larger penetration depth, related to an increase in reflectance in this region (Figure 1a), corresponding to a decrease of the effective absorptance; this observation is consistent with similar trends reported for related metal oxide semiconductors.^6,14,15,22,23,25,26,40^ This phenomenon either reflects a decrease of the absorption coefficient, or complications determining absorption due to combined effects of surface reflectivity, optical interference within the film attributed to the porous nature of the material and surface roughness. For the experiments reported here, the *LHE* and penetration depth can be considered to be similar for the three LEDs used. The increase in *LHE* at wavelengths larger than 550 nm corresponds to absorption to midgap states, which is further addressed in the discussion of Figure 10.

Energy band gap values were estimated using Kubelka–Munk analysis^40,41^ (Figure S5) from the DRS data on the five film samples, presenting similar values for Mg_0.1_Cu_0.9_V_2_O_6_, Ca_0.1_Cu_0.9_V_2_O_6_ and CuV_2_O_6_. The absence of Cu in the pure CaV_2_O_6_ and MgV_2_O_6_ samples greatly enlarges the band gap of these photoelectrodes, resulting in a much lower *LHE*. The DRS data on the five film samples are in good agreement with the trends presented in our earlier study^14^ on the corresponding SCS-derived powder samples.

### 3.2 Photoelectrochemical (PEC) Measurements

Figure 2 shows linear sweep voltammetry (LSV) curves for CuV_2_O_6_, Mg_0.1_Cu_0.9_V_2_O_6_ and Ca_0.1_Cu_0.9_V_2_O_6_ photoelectrodes in 0.1 M phosphate buffer as electrolyte at a potential scan rate of 20 mV s^–1^ with chopped front side illumination using different light sources: 1 sun AM 1.5G, UV (λ = 370 nm), blue (λ = 455 nm) and green (λ = 535 nm) illumination, at the same LED photon flux of 1.6 × 10^16^ cm^–2^ s^–1^. The photocurrent generated by CaV_2_O_6_ and MgV_2_O_6_ electrodes was very small, hence, these electrodes were excluded from further analysis. Also, measurements under red LED illumination resulted in negligible photocurrent for all electrodes. The photocurrent onset was located at 1.0 V vs RHE, and the CuV_2_O_6_ film exhibited the highest photocurrent, reaching 0.26 mA cm^–2^ at 1.8 V vs RHE compared to 0.17 mA cm^–2^ and 0.15 mA cm^–2^ for Ca_0.1_Cu_0.9_V_2_O_6_ and Mg_0.1_Cu_0.9_V_2_O_6_, respectively. The disparity in photocurrent decreased with increasing wavelength, with the CuV_2_O_6_ electrode achieving twice the photocurrent under UV illumination, 1.5 times higher under blue light and comparable performance under green light conditions in comparison with the two alloy vanadates. This behavior is not correlated with the light penetration depth, where the larger penetration depth in the UV region corresponds to lower light absorption. A possible explanation for this phenomenon will be provided in the following section. On the other hand, the higher photocurrent observed for the CuV_2_O_6_ film can be partially attributed to the higher *LHE*, but also implies better kinetic properties in comparison with the alloy modifications.

**Figure 2:**
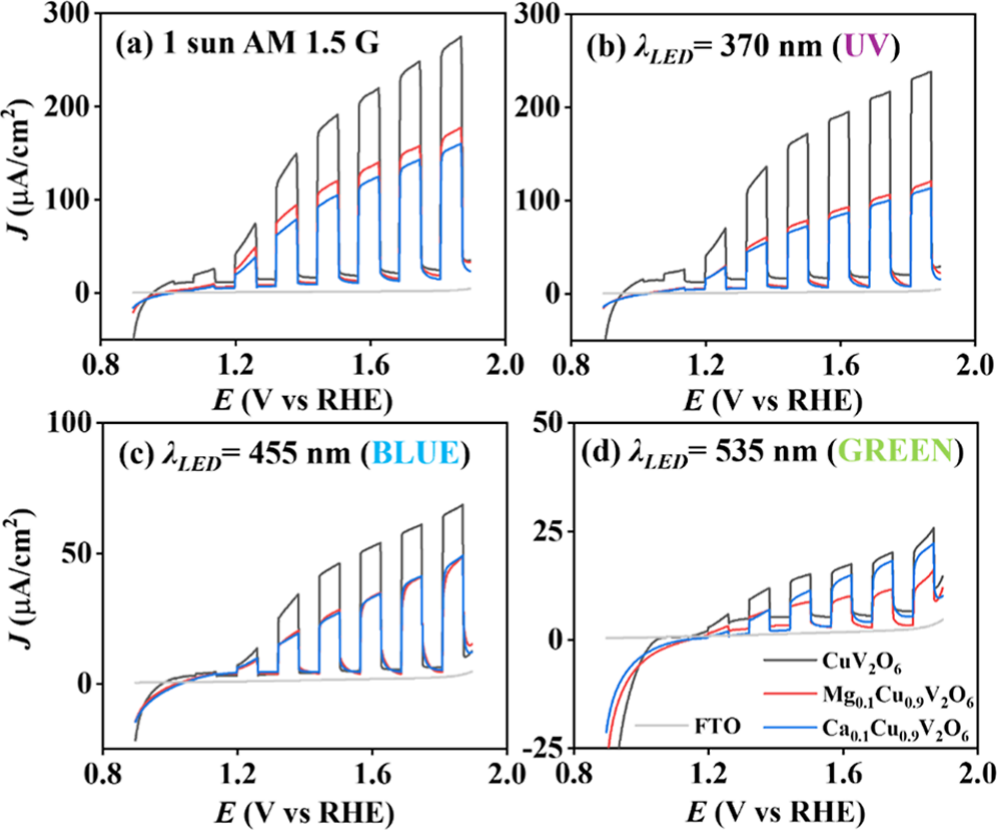
LSV curves with chopped front side illumination at (a) 1 sun AM 1.5G (b) λ_LED_ = 370 nm (UV), (c) λ_LED_ = 455 nm (blue) and (d) λ_LED_ = 535 nm (green) at the same photon flux of 1.6 × 10^16^ cm^–2^ s^–1^ in 0.1 M phosphate buffer as electrolyte with a potential scan rate of 20 mV s^–1^ for CuV_2_O_6_, Mg_0.1_Cu_0.9_V_2_O_6_ and Ca_0.1_Cu_0.9_V_2_O_6_ photoanodes screen-printed on FTO.

Figure S6 shows LSV curves under chopped light with 1 sun AM 1.5G filter as light source with 0.1 M phosphate buffer and 0.1 M Na_2_SO_3_ as hole scavenger for CuV_2_O_6_, CaV_2_O_6_, MgV_2_O_6_, Ca_0.1_Cu_0.9_V_2_O_6_ and Mg_0.1_Cu_0.9_V_2_O_6_ electrodes. Except for the CaV_2_O_6_ film, a large current corresponding to the oxidation of sulfite was observed even in the dark, which is attributed to direct charge transfer from the FTO, due to only partial coverage of the FTO by the metal oxide material. Hence, this system is unsuitable for the study of photogenerated hole transfer kinetics and was not further explored.

The potential distribution at the semiconductor–electrolyte solution interface and interfacial charge transfer kinetics play a crucial role in photocurrent generation, directly influencing the efficiency of charge separation, transport, charge transfer and recombination in the material. It should be kept in mind that water oxidation to oxygen involves the transfer of 4 holes and 4 protons, and the interfacial kinetics are expected to be very complex. Recent work on photoelectrochemical water oxidation at hematite illustrates these aspects, detailing the challenges to determine the reaction mechanism, the molecularity and reaction order of the rate-determining step, light intensity dependence, band edge unpinning and interfacial chemistry.^42^ Charge transfer therefore does not simply correspond to hole transfer to the solution, but rather involves interfacial reactions at the semiconductor surface.

Carrier recombination may occur in the bulk or at the surface on localized states that may correspond to reaction intermediates or even charge transport states. Hence, the competition between charge transfer, recombination and charge transport depends on many factors. Therefore, when detailed information on the mechanisms cannot be accessed, it is preferred to use a simple model. The most generally used model considers that charge separation competes with bulk recombination on a short time scale, while surface recombination competes with charge transfer at longer times.^33^ In addition, it is often assumed that charge transfer occurs via surface states corresponding to reaction intermediates, which also act as recombination centers.^33,43^ Note that for porous, mesoscopic photoelectrodes the potential distribution is expected to be significantly different from classical single crystal semiconductors, which affects the driving force for charge separation and transport, thus complicating the interpretation of experimental results.^35^

To gain a more comprehensive understanding of these processes, a concurrent use of (P)­EIS and IMPS techniques^17,19^ was employed in this study. As discussed below, these complementary techniques provided valuable insights into the dynamic behavior of charge carriers under operational conditions, enabling a detailed analysis of the factors affecting efficiency loss. In (P)­EIS, the modulated applied potential was applied to the FTO substrate, and the partial coverage of the FTO indicated that the total impedance contained contributions from both the semiconductor–electrolyte solution and the FTO–electrolyte solution interface. On the other hand, the modulated light intensity used in IMPS was only absorbed in the semiconductor. Thus, the effect of uncovered FTO may be smaller, providing an additional and complementary analysis of this complex system.

### 3.3 (Photo)­Electrochemical Impedance Spectroscopy

Figure 3 shows the (P)­EIS plots as a function of applied potential under dark and blue (λ = 455 nm) LED illumination at a photon flux of 1.6 × 10^16^ cm^–2^ s^–1^ in 0.1 M phosphate buffer as electrolyte for FTO, CuV_2_O_6_, Mg_0.1_Cu_0.9_V_2_O_6_ and Ca_0.1_Cu_0.9_V_2_O_6_. The Nyquist graphs presented similar characteristics in all cases, with one loop dominating the upper quadrant. On the other hand, the Mg_0.1_Cu_0.9_V_2_O_6_ and Ca_0.1_Cu_0.9_V_2_O_6_ electrodes showed a second arc in the dark (refer to the Bode plots in Figure S7). Hence, surprisingly, the complex photoelectrode impedance can be characterized by one time constant for most cases; two time constants are only needed for the doped systems in the dark.

**Figure 3:**
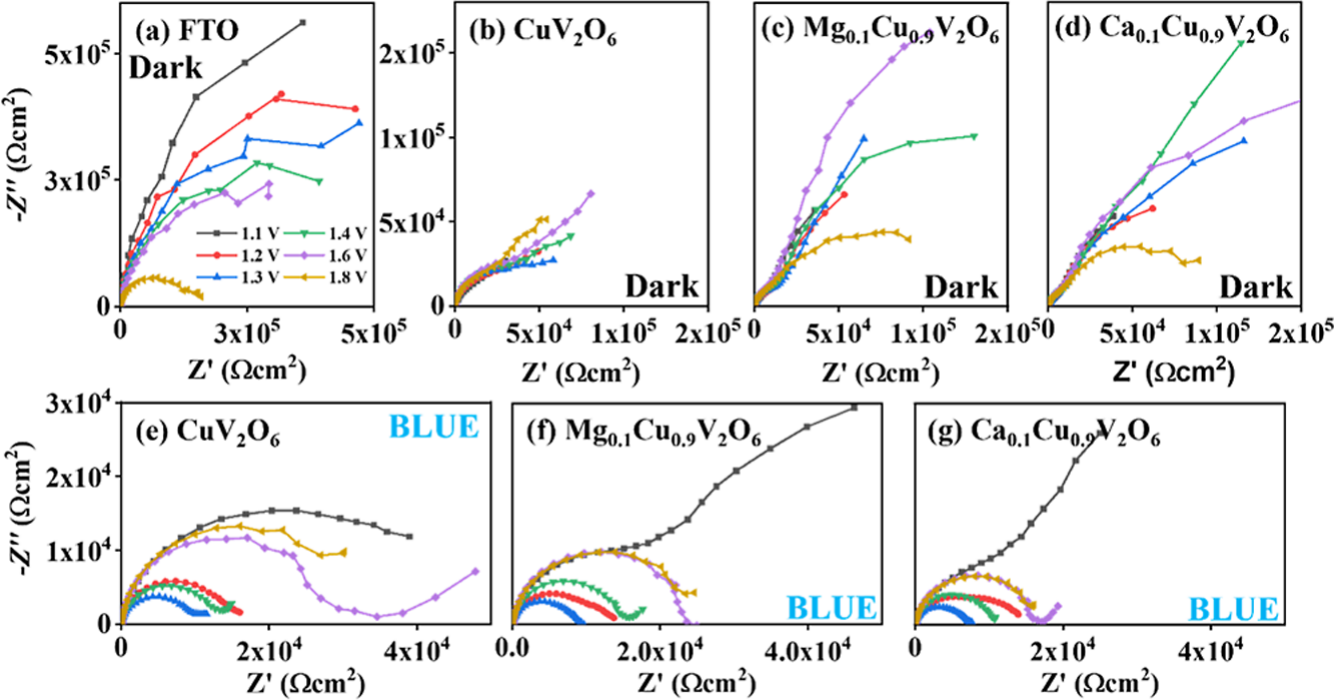
(P)­EIS data plotted in Nyquist format at different applied potentials under (a–d) dark and (e–g) blue (λ = 455 nm) LED illumination at photon flux of 1.6 × 10^16^ cm^–2^ s^–1^ in 0.1 M phosphate buffer as electrolyte for (b,e) CuV_2_O_6_, (c,f) Mg_0.1_Cu_0.9_V_2_O_6_ and (d,g) Ca_0.1_Cu_0.9_V_2_O_6_ photoelectrodes screen-printed on FTO. The data for the FTO substrate as reference is shown in frame (a).

Figure S8 shows an equivalent circuit that aims to illustrate the complexity of the photoelectrode electrical properties related to the morphology of the porous metal oxide film, considering that the FTO substrate is also in direct contact with the electrolyte solution. The FTO–electrolyte interface impedance appears in parallel to that corresponding to the metal oxide–electrolyte interface, and may be represented by a simple circuit, with an effective capacitance corresponding to the FTO capacitance in series with the Helmholtz capacitance at the electrolyte side, in parallel to a resistance that describes charge transfer.

The metal oxide–electrolyte impedance generally needs to be represented by a complex equivalent circuit, where the semiconductor capacitance is expected to be smaller than that of the Helmholtz layer, and additional processes involving electronic surface states that may also act as recombination centers and charge transfer states need to be considered.^43^ In addition, if trap-limited charge transport within the semiconductor affects the total impedance, a transmission line impedance is often used to model the system. However, if only one time constant (one semicircle) is observed in the EIS spectra, this complex total circuit is modeled by a reduced equivalent circuit shown in Figure S8b: the total capacitance contains contributions from the semiconductor, possibly including interfacial trap states, and the Helmholtz layer. The total parallel resistance comprises the resistances corresponding to generation, transport, trapping, interfacial charge transfer, and is proportional to the potential-dependent inverse slope of the *J*–*E* curve.

The series resistance (*R*_s_) obtained from fitting the spectra to these circuits, shown in Figure S9a, was found to be essentially independent of material, and the same in the dark and under illumination, and was principally dominated by the FTO substrate. Figure S10 illustrates the quality of the fits at a representative potential (1.8 V vs RHE) under dark and light conditions, and the extracted impedance parameters along with the errors are summarized in Table S1 of the Supporting Information.

Figure 4 shows the parallel resistance, corresponding to *R*_p,TOT_ for the spectra with one arc, and *R*_p,1_ or *R*_p,2_ for the results with two arcs (Figure S8a), and the effective capacitance (*C*_eff_) under dark and blue illumination conditions for FTO (as a reference), CuV_2_O_6_, Mg_0.1_Cu_0.9_V_2_O_6_ and Ca_0.1_Cu_0.9_V_2_O_6_ screen-printed onto FTO. The largest difference in *R*_p_ obtained from the high-frequency loop between dark and light is observed for the CuV_2_O_6_ photoelectrode, which is in concordance with the results observed in Figure 2. In the dark, *R*_p_ was essentially independent of potential, while under illumination *R*_p_ first decreased when changing the applied potential in the positive direction up to 1.3 V vs RHE, coinciding with the observation of the onset and increase of the photocurrent. Hence, in this potential regime, the parallel resistance is determined by the charge transfer resistance and its decrease translates into a larger photocurrent.^44–47^ At more positive potential, *R*_p_ increased again, in agreement with the decreasing slope of the *J*–*E* curve reaching a saturated photocurrent. In this regime, the photocurrent was limited by the balance between generation, recombination and charge transfer. Although the charge transfer resistance likely no longer dominated the total parallel resistance, slower charge transfer kinetics could also occur due to surface poisoning. Phosphate groups adsorbed to the photoelectrode surface are capable of passivating the catalytically active sites or reaction intermediates, thereby limiting hole transfer.^51,52^ Support for this hypothesis is provided by the FTIR–ATR spectra for films before and after PEC measurement (Figure S11), where new bands in the 1300 to 900 cm^–1^ regions appeared exclusively for the photoelectrochemically treated films. These bands are consistent with phosphate groups either directly adsorbed onto the surface or bound to surface hydroxyls to form phosphorylated –OH species.^53,54^

**Figure 4:**
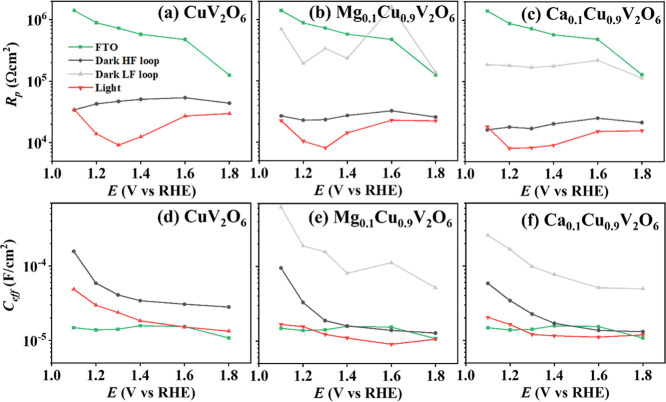
(a–c) Values for *R*_p_ and (d–f) *C*_eff_ extracted from the (P)­EIS data at different applied potentials under dark and light conditions with blue (λ = 455 nm) LED at a photon flux of 1.6 × 10^16^ cm^–2^ s^–1^ in 0.1 M phosphate buffer as electrolyte for FTO as reference and (a,d) CuV_2_O_6_, (b, e) Mg_0.1_Cu_0.9_V_2_O_6_ and (c,f) Ca_0.1_Cu_0.9_V_2_O_6_ electrodes screen-printed on FTO.

On the other hand, the charge transfer resistance obtained from the low-frequency loop observed for Mg_0.1_Cu_0.9_V_2_O_6_ and Ca_0.1_Cu_0.9_V_2_O_6_ was very similar to that for bare FTO and can be attributed to the photoelectrode morphology. In this case, the FTO–electrolyte interface impedance is observed as a second arc at low frequencies.

The effective capacitance (*C*_eff_) for bare FTO in contact with the electrolyte was about 15 μF/cm^2^, in agreement with the expected value of the Helmholtz capacitance (*C*_H_).^48^ The capacitance of the photoelectrodes was larger under dark conditions in the potential range up to 1.4 V vs RHE, which indicates that the larger surface area for the Mg_0.1_Cu_0.9_V_2_O_6_ and Ca_0.1_Cu_0.9_V_2_O_6_ electrodes contributes to the differential capacitance. At more positive potentials, the capacitance was very similar to that of FTO implying the mixed oxides are depleted. For the CuV_2_O_6_ photoelectrode, the capacitance in the dark remains larger than for FTO, indicating that the surface remains in electronic contact with the FTO substrate.^49,50^ Under illumination, the capacitive properties follow a similar trend; however, the effective capacitance is generally smaller than in the dark and closer to the value obtained for FTO. Combined, these results indicate that the impedance of the semiconductor is smaller under illumination.

### 3.4 Intensity-Modulated Photocurrent Spectroscopy (IMPS)

Figure 5 shows the IMPS spectra as a function of the applied potentials under front side illumination using three LEDs: UV (λ = 370 nm), blue (λ = 455 nm) and green (λ = 535 nm) with the photon flux constant at 1.6 × 10^16^ cm^–2^ s^–1^ for CuV_2_O_6_, Mg_0.1_Cu_0.9_V_2_O_6_ and Ca_0.1_Cu_0.9_V_2_O_6_ photoanodes in a 0.1 M phosphate buffer electrolyte. The Nyquist-type plot of the CuV_2_O_6_ electrode was dominated principally by one loop in the fourth quadrant, with a characteristic frequency at the minimum (*f*_min_). For the Mg_0.1_Cu_0.9_V_2_O_6_ and Ca_0.1_Cu_0.9_V_2_O_6_ electrodes, at applied potentials more positive than 1.4 V vs RHE, a second, loop appeared in the fourth quadrant. The size of this second loop increased with more positive applied potential. Interestingly, the second loop was absent in the CuV_2_O_6_ film for any tested wavelength. It was also absent for Mg_0.1_Cu_0.9_V_2_O_6_ and Ca_0.1_Cu_0.9_V_2_O_6_ films under UV illumination. However, it did appear and develop upon increasing the wavelength; the frequency at the minimum of this second arc, *f*_min2_, is observed at lower frequency than *f*_min_.

**Figure 5:**
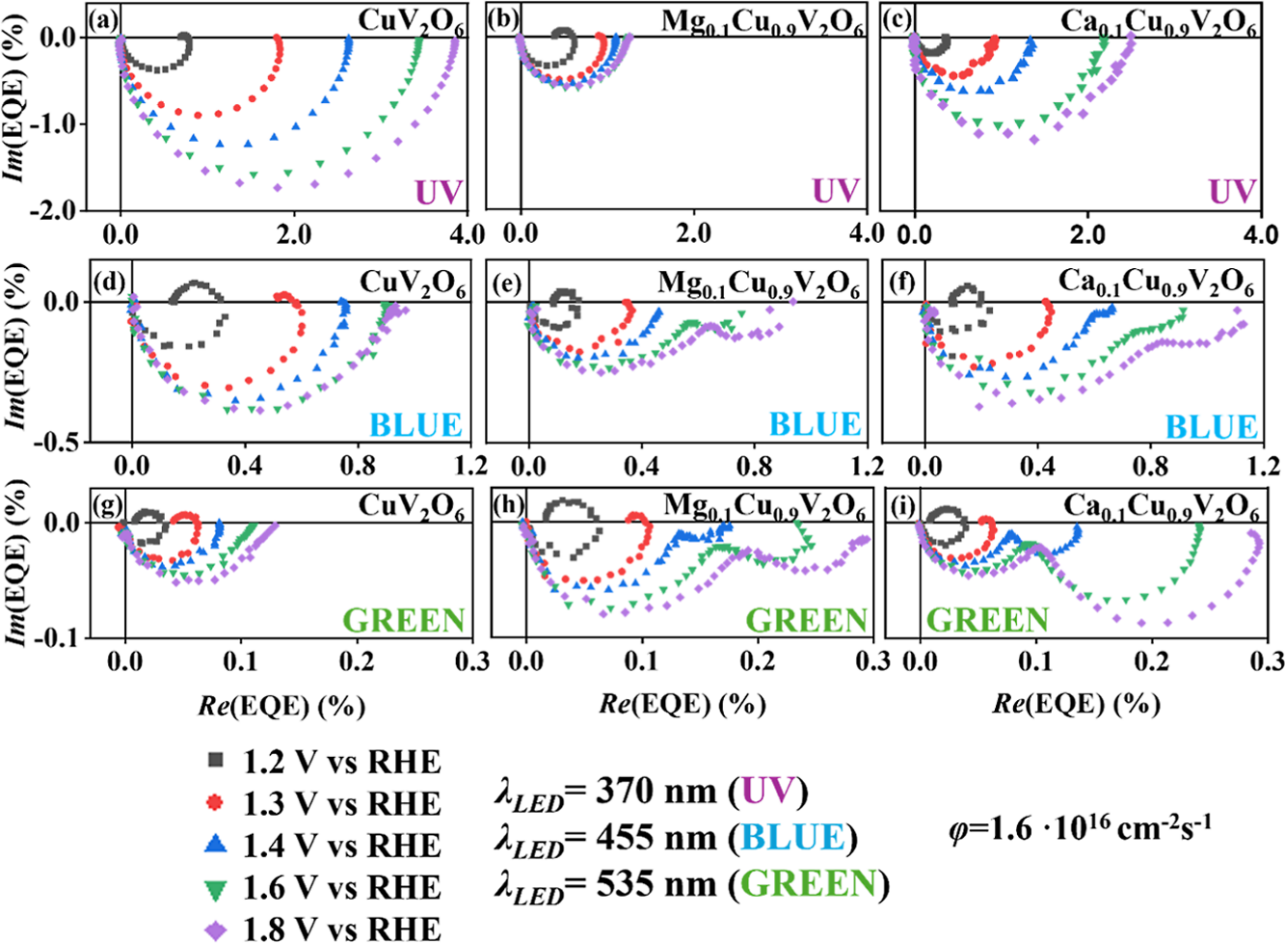
IMPS data in Nyquist plot format at different applied potentials under different wavelength LED illumination (a–c) UV λ = 370 nm, (d–f) blue λ = 455 nm, (g–i) green λ = 535 nm, at the same photon flux 1.6 × 10^16^ cm^–2^ s^–1^ in front side illumination with 0.1 M phosphate buffer as electrolyte for (a,d,g) CuV_2_O_6_, (b,e,h) Mg_0.1_Cu_0.9_V_2_O_6_ and (c,f,i) Ca_0.1_Cu_0.9_V_2_O_6_ electrodes screen-printed on FTO.

The high-frequency loop in IMPS spectra is generally attributed to the cell’s *RC* time constant, which is determined by the total (effective) capacitance and the series cell resistance.^31,32^ For a photoelectrode on FTO, the series resistance would correspond to the FTO substrate, while the total capacitance would be determined by the semiconductor (space charge) capacitance, the surface state capacitance and the Helmholtz layer capacitance. Figure S9b shows the capacitance versus applied potential calculated from *C* = (2π *f*_min_ *R*_s_)^−1^, where *R*_s_ was obtained from the EIS measurement (shown in Figure S9a). It can be observed that the capacitance increases upon shifting the applied potential from 1.2 to 1.4 V, and it saturates at about 15 μF cm^–2^ at more positive potentials. This relatively high value indicates that the semiconductor capacitance is similar in magnitude to that of the Helmholtz layer. This also implies that the applied potential is distributed between the semiconductor and the Helmholtz layer, resulting in a shift of the band edges upon changing the applied potential, i.e. band edge unpinning. The initial increase of the capacitance from 1.2 to 1.4 V vs RHE could indicate a buildup of hole density at the interface upon shifting the applied potential positively, but may also result from surface recombination as observed at lower frequencies in this potential range (see below).

For the high-frequency loop, in absence of a second loop in the fourth quadrant, the intersection with the real axis corresponds to the maximum *EQE*, excluding surface processes losses (i.e., unattenuated *EQE*), which increased with more positive applied potential. The *EQE* and therefore, the charge separation efficiency, decreased with larger wavelengths, reflecting the behavior observed above in the current–potential curves shown in Figure 2. At applied potentials more negative than 1.3 V vs RHE, a low-frequency loop appeared in the first quadrant, where the frequency of the maximum reflects the competition between the hole transfer and surface recombination, according to the simple model where electrons in the conduction band may still reach the surface to recombine with (trapped) holes. However, this loop quickly disappeared at more positive potential, indicating that either fast electron extraction at the FTO interface or the internal field prevents surface recombination at these potentials. Moreover, it did not reappear under varying illumination intensity or for different wavelengths.

Consequently, the rate constants for charge transfer (*k*_tr_) and surface recombination (*k*_rec_) cannot be determined at more positive potentials. Notably, this behavior is consistent with the photocurrent transients under chopped illumination (see Figure 2), which do not exhibit significant overshoot and delays, indicating minimal surface recombination.^33^ These observations suggest that the photoresponse is not limited by *surface* recombination processes.

Figure 6 presents the IMPS spectra as a function of photon flux using different LEDs under front side illumination: UV (λ = 370 nm), blue (λ = 455 nm) and green (λ = 535 nm), at a constant applied potential of 1.8 V vs RHE for CuV_2_O_6_, Mg_0.1_Cu_0.9_V_2_O_6_ and Ca_0.1_Cu_0.9_V_2_O_6_ photoanodes.

**Figure 6:**
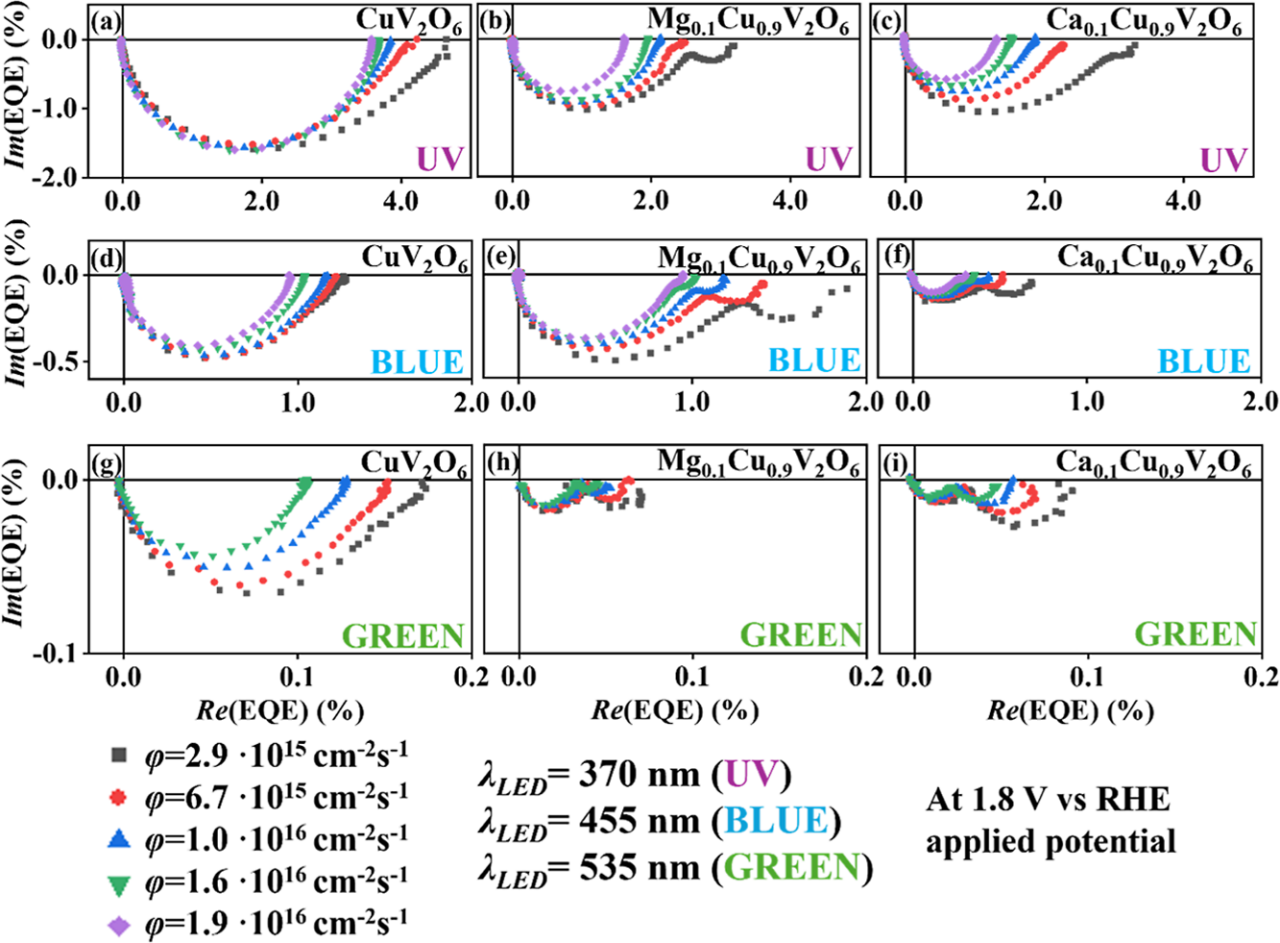
IMPS data in Nyquist plot format at varying photon flux under different wavelength LED illumination (a–c) UV λ = 370 nm, (d–f) blue λ = 455 nm, (g–i) green λ = 535 nm, at a fixed applied potential of 1.8 V vs RHE in 0.1 M phosphate buffer as electrolyte for (a,d,g) CuV_2_O_6_, (b,e,h) Mg_0.1_Cu_0.9_V_2_O_6_ and (c,f,i) Ca_0.1_Cu_0.9_V_2_O_6_ films screen-printed on FTO.

The *EQE* at the intersection on the real axis at low frequency decreased with increasing photon flux, indicating that efficiency losses due to bulk recombination become more significant at higher light intensities, i.e., larger charge densities. Figure S12 shows a sublinear intensity dependence with a slope of about −0.5 for the Ca- and Mg-containing CuV_2_O_6_ samples. Similar sublinear trends have been reported in solar cells, where this observation is associated with the recombination mechanism.^55–59^ Within this interpretation, the slope of about −0.5 points to second-order (bimolecular) recombination kinetics for the Ca- and Mg-containing samples. In contrast, the pure CuV_2_O_6_ material shows a much weaker intensity dependence, closer to first-order (monomolecular) kinetics.

Once again, the second semicircle was absent for CuV_2_O_6_ film under any photon flux tested. In contrast, when the photon flux was sufficiently low, the magnesium and calcium copper vanadate electrodes developed a second arc, even under UV illumination. This second loop decreased as the light intensity increases.

Figure 7 shows *f*_min_ as a function of (Figure 7a–c) of the applied potential at the same photon flux of 1.6 × 10^16^ cm^–2^ s^–1^ and varying photon flux (Figure 7d–f) at an applied potential of 1.8 V vs RHE, under different wavelength LED illumination for CuV_2_O_6_, Mg_0.1_Cu_0.9_V_2_O_6_ and Ca_0.1_Cu_0.9_V_2_O_6_ electrodes, respectively. The *f*_min_ value was similar for all samples and was independent of the illumination wavelength and the external bias, except for the applied potential below 1.3 V vs RHE, which was influenced by the loop in the first quadrant in the Nyquist plot. In contrast, *f*_min_ showed a weak dependence on the photon flux and increased with increasing light intensity. There are several possible explanations for this observation, including the slight decrease of *R*_p_ under illumination. On the other hand, a similar light intensity dependence of *f*_min_ has been previously reported for nanostructured, mesoscopic TiO_2_ and WO_3_ photoelectrodes, among other materials. The observed dependence is generally attributed to trap-limited charge transport: as the light intensity increases, deeper traps are filled up, leading to shallower traps to govern the transport kinetics.^21,27,60–65^ This effect is well-known in dye-sensitized solar cells, but it is not exclusive to nanostructured systems. Recent studies have demonstrated that trap-limited transport also occurs in bulk metal oxide photoanodes with larger particle sizes, such as BiVO_4_.^66,67^

**Figure 7:**
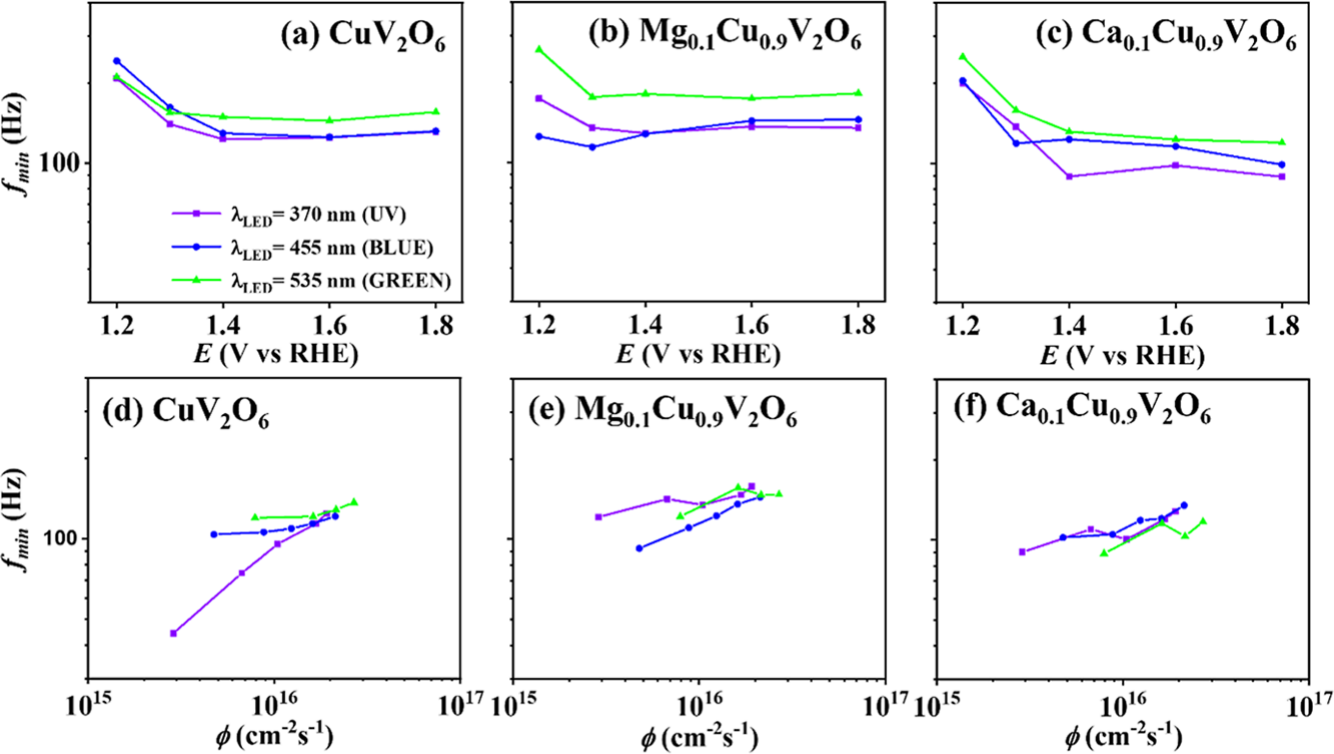
IMPS parameter *f*_min_ under different wavelength LED illumination, with 0.1 M phosphate buffer as electrolyte for (a–c) different applied potentials at the same photon flux 1.6 × 10^16^ cm^–2^ s^–1^ and for (d–f) different photon fluxes at an applied potential of 1.8 V vs RHE for (a,d) CuV_2_O_6_, (b,e) Mg_0.1_Cu_0.9_V_2_O_6_ and (c,f) Ca_0.1_Cu_0.9_V_2_O_6_ photoelectrodes screen-printed on FTO.

Figure 8 shows the parameter *f*_min2_ as a function of applied potential (Figure 8a,b) at a fixed photon flux of 1.6 × 10^16^ cm^–2^ s^–1^ and varying photon flux (Figure 8c,d) at an applied potential of 1.8 V vs RHE, under different wavelength LED illumination for Mg_0.1_Cu_0.9_V_2_O_6_ (Figure 8a,c) and Ca_0.1_Cu_0.9_V_2_O_6_ (Figure 8b,d) electrodes. The values of *f*_min2_ were similar for both electrodes and did not depend on the applied potential. On the other hand, *f*_min2_ decreased with increasing wavelength and depended strongly on the photon flux, increasing rapidly with increasing light intensity.

**Figure 8:**
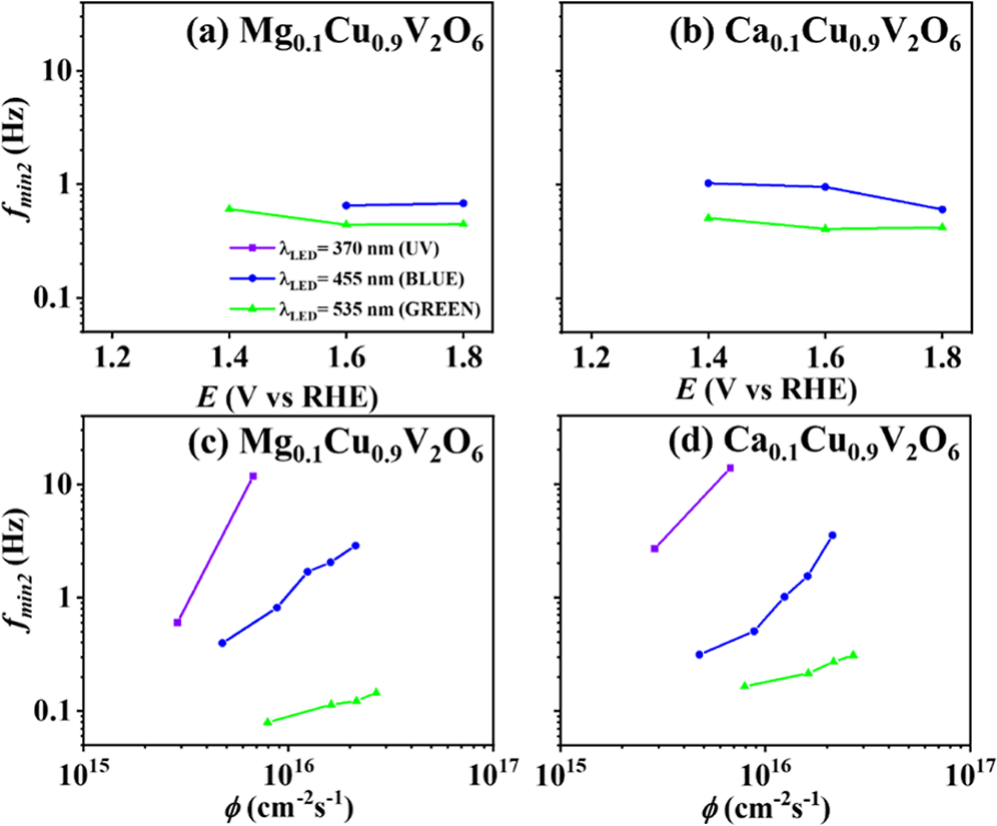
*f*_min2_ under different wavelength LED illumination with 0.1 M phosphate buffer as an electrolyte at (a,b) different applied potentials at the same photon flux 1.6 × 10^16^ cm^–2^ s^–1^ and at (c,d) different photon fluxes at an applied potential of 1.8 V vs RHE for (a,c) Mg_0.1_Cu_0.9_V_2_O_6_ and (b, d) Ca_0.1_Cu_0.9_V_2_O_6_ photoanodes screen-printed on FTO. Note that a second loop was not observed for CuV_2_O_6_, which was also the case for several potentials and light intensities for the modified materials, hence *f*_min2_ could not be determined under these conditions.

A second loop in the fourth quadrant has been shown to occur when the space charge and Helmholtz capacitance are of similar magnitude, with the total capacitance given by *C*_SC_ *C*_H_/(*C*_SC_ + *C*_H_), and the charge transfer constant becomes larger than the surface recombination constant;^31,32^ this may be the case upon shifting the potential more positive, in the presence of a hole scavenger, or at larger photon flux.

The accumulation of photogenerated holes at higher light intensities near the surface may induce a modification in the potential drop across the Helmholtz layer, which may result in an increase of the hole transfer rate constant. This interpretation is consistent with prior work on light-induced modulation of interfacial potentials in photoelectrochemical systems.^42,68–70^ Interestingly, the second loop is only observed for the Mg- and Ca-modified copper vanadates, indicating modifications in the electronic structure upon partial substitution of Cu^2+^ with Mg^2+^ or Ca^2+^. This may affect Cu–O hybridization and lead to the formation of oxygen vacancies, which can act as trapping or recombination centers for charge carriers.^14,15,71^ These defects increase the charge transfer kinetics to the solution, resulting in the second loop in the IMPS spectra; however, the DC photocurrent is actually smaller than for copper vanadate, indicating that the charge separation efficiency is smaller due to enhanced (bulk) recombination as confirmed by the smaller high-frequency loop.

### 3.5 Putting the (P)­EIS and IMPS Data Together

The characteristic frequencies of the different interfacial processes considered in this paper are summarized in Figure 9. Under dark conditions, the low-frequency loop in the EIS spectra of magnesium and calcium vanadate samples was attributed to the FTO substrate. In contrast, under illumination, the inverse time constant extracted from PEIS matched with the low-frequency loop characterized by *f*_min2_ in IMPS measurement, under the same conditions. Hence, the low-frequency time constant from IMPS and the time constant in PEIS describe the charge transfer kinetics at the photoelectrode interface. IMPS results in additional information on the potential distribution and total capacitance in the high frequency loop. Furthermore, the surface recombination feature observed in the first quadrant of IMPS spectra in the potential range of 1.2 to 1.4 V vs RHE cannot be distinguished in the PEIS measurements, indicating the complementary character of the two small-signal modulation techniques.

**Figure 9:**
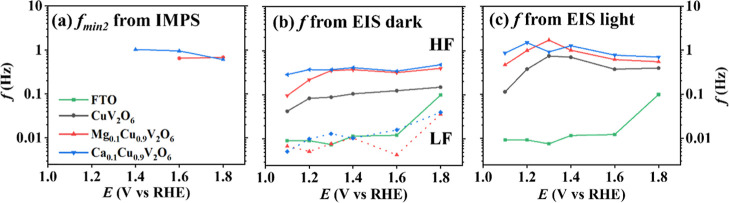
Frequencies extracted from (a) IMPS (b) EIS in dark conditions and (c) PEIS under illumination. The photon flux was fixed at 1.6 × 10^16^ cm^–2^ s^–1^ with BLUE (λ = 455 nm) LED in 0.1 M phosphate buffer as electrolyte for FTO as reference, CuV_2_O_6_, Mg_0.1_Cu_0.9_V_2_O_6_, and Ca_0.1_Cu_0.9_V_2_O_6_ films screen-printed on FTO.

Finally, the two parameters *LHE* and the *EQE* , of critical importance to practical applications of these materials (e.g., energy conversion), as extracted from chronoamperometry and IMPS data, may be compared. This is shown in Figure 10 as a function of wavelength for CuV_2_O_6_, Mg_0.1_Cu_0.9_V_2_O_6_ and Ca_0.1_Cu_0.9_V_2_O_6_ photoelectrodes at a potential of 1.8 V vs RHE and the same photon flux of 1.6 × 10^16^ cm^–2^ s^–1^ for each LED. Although the *LHE* increases up to 400 nm and remains constant until 600 nm, both *EQE*, extracted from chronoamperometry and from IMPS, decreased with increasing wavelength, which mirrors the behavior observed in Figure 1.

**Figure 10:**
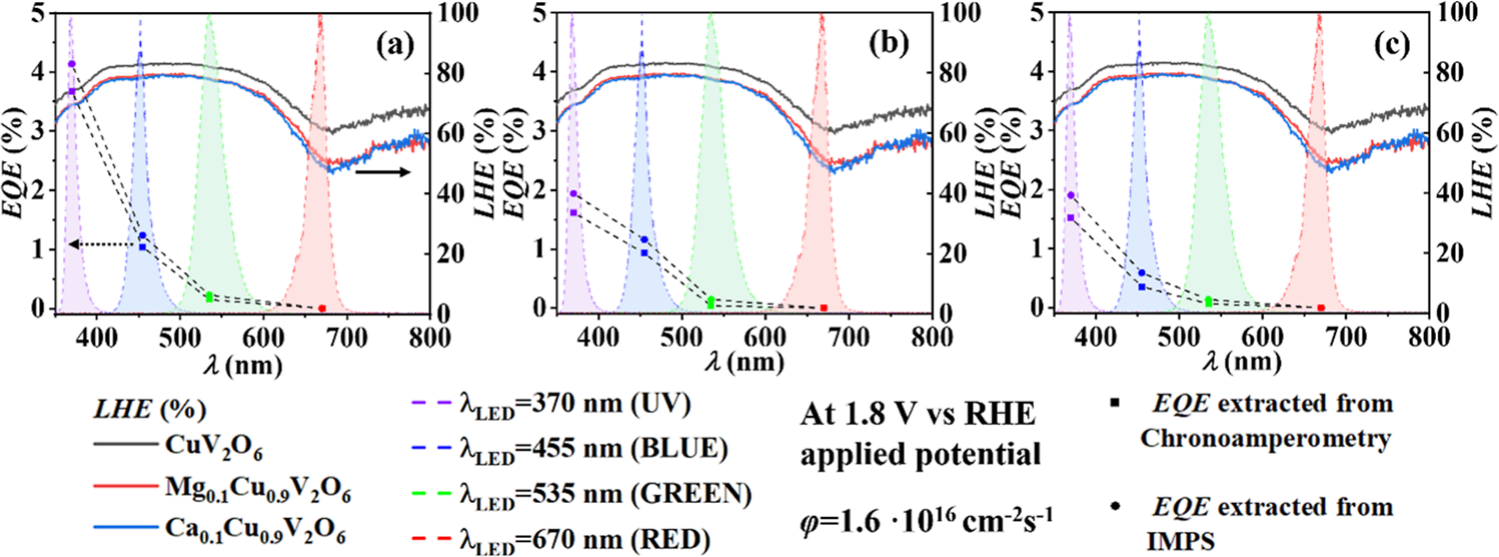
*LHE* and *EQE* extracted from chronoamperometry (circles) and IMPS (squares) vs illumination wavelength for (a) CuV_2_O_6_, (b) Mg_0.1_Cu_0.9_V_2_O_6_, and (c) Ca_0.1_Cu_0.9_V_2_O_6_ screen-printed on FTO, at an applied potential of 1.8 V vs RHE and photon flux of 1.6 × 10^16^ cm^−2^ s^−1^. The emission spectrum of each used LED in the PEC measurements is represented by dashed lines.

This observation is attributed to the electronic band structure of α-CuV_2_O_6_.^15^ The top of the valence band is primarily composed of strongly hybridized Cu 3d–O 2p orbitals, whereas the bottom of the conduction band is dominated by V 3d states with a minor contribution from O 2p orbitals. At shorter wavelengths (UV illumination), the photon energy is sufficiently high to drive direct transitions from the valence band to the conduction band, leading to efficient charge carrier generation and, consequently, a high *EQE*. In contrast, at larger wavelengths, even though the material continues to absorb light (as indicated by the constant *LHE* ), the lower photon energies are insufficient to effectively promote electrons via a direct optical transition. Instead, any excitation involves the midgap states, primarily Cu 3d e_g_ states hybridized with O 2p, which require an O-p to O-p transition that is intrinsically less favorable^14–16,25^ This results in a reduced efficiency in generating free carriers and can account for the observed decrease in *EQE* with increasing wavelength.

We note that our present observation (Figures 1 and 10) that a significant portion of the absorbed photons do not generate mobile charge carriers seems to be prevalent for several metal oxide semiconductors as recently reviewed by other authors.^24^ Interestingly, this group indeed includes copper vanadates as well (cf., see Figure 2 cited in their review), and four separate sets of data on declining *EQE* (or *IQE*) vs wavelength for copper vanadates were cited by these authors.^24^ This alloying strategy was motivated by the potential of alkaline earth metals, such as Mg and Ca, to enhance water adsorption properties and alter the electronic structure in a way that could reduce recombination losses, thereby improving photocarrier generation and injection efficiency.^14–16^ However, the optimistic projection by these authors^24^ that photocarrier generation yield losses can be possibly mitigated by alloying does not seem to be borne out by the trends in this study on alloying Cu with either Mg or Ca within the vanadate compound framework.

Nevertheless, the results presented in this work provide valuable insights into how alkaline earth substitution affects the optical and interfacial properties of multinary vanadates. These findings contribute to a broader understanding of the challenges in designing efficient photoelectrodes and highlight the need for further exploration of compositional tuning strategies in complex metal oxides.

## 4 Conclusions

The combined use of IMPS and (P)­EIS in this study has furnished useful insights into the dynamic consequences of partially or wholly replacing copper with an alkaline earth metal (Mg, Ca) in a metavanadate compound matrix (MV_2_O_6_). Our new data on these compounds and alloys also show that the phenomenon of nonunity photogeneration yield of mobile charge carriers pervades many transition metal oxide semiconductors (both binary and ternary compounds) and in this sense, is more prevalent than may have been envisioned. Indeed, the notion of optoelectronic behavior akin to “bound excitons” may be evoked for photocarriers generated from localized optical transitions of the d–d type. Mitigating this trend and improving *EQE* (or *IQE*), and thereby the overall photoconversion efficiencies of devices based on these materials, would require research beyond the scope of the present study.

## Supplementary Material



## Notes

Photographs of screen-printed layers on FTO; PXRD data on the five thin film samples; top view and cross-section SEM images; DRS data and Kubelka–Munk plots; *J*–*E* curves under chopped light irradiation; EIS data in Nyquist and Bode formats; equivalent circuit model for the (P)­EIS data fit; ATR–FTIR data (PDF)
