# Supplementary material for: Charge Dynamics as Probed by Small Signal-Modulated Photocurrent and Impedance Spectroscopy of Metal Vanadate Semiconductors and Alloys
Source: ACS Appl Opt Mater. 2025 Jul 25;3(8):1684–95. doi: 10.1021/acsaom.5c00154 (PMC12697059; doi:10.1021/acsaom.5c00154)
Supplement: Supplementary file 1 [file ot5c00154_si_001.pdf]

Supporting Information

Charge Dynamics as Probed by Small Signal-Modulated Photocurrent and Impedance Spectroscopy of Metal Vanadate Semiconductors and Alloys

Juan Carlos Expósito-Gálvez[1], Abhishek Rawat[2], Krishnan Rajeshwar[2*], Gerko Oskam[1*]

[1]*Center for Nanoscience and Sustainable Technologies (CNATS). Department of Physical, Chemical and Natural Systems, Universidad Pablo de Olavide, Sevilla, 41013, Spain.*

[2]*Department of Chemistry & Biochemistry, The University of Texas at Arlington, Arlington, TX-76019, USA.*

Corresponding Authors

***E-mail:** gosk@upo.es*

rajeshwar@uta.edu.

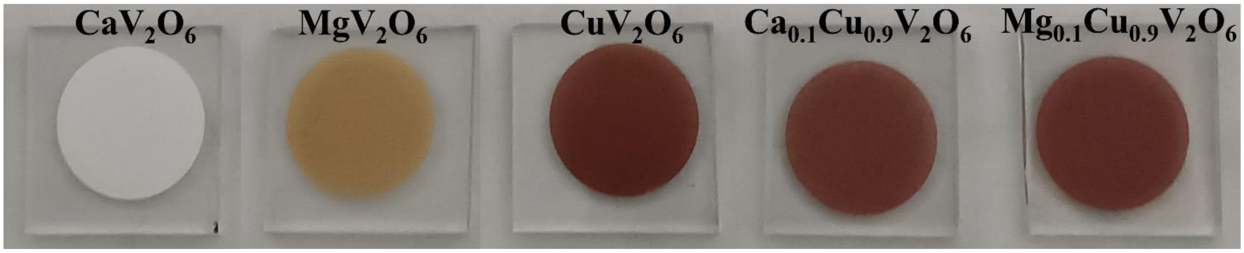

Figure S1. Photos of 5-layer films deposited on FTO using the screen-printing technique.

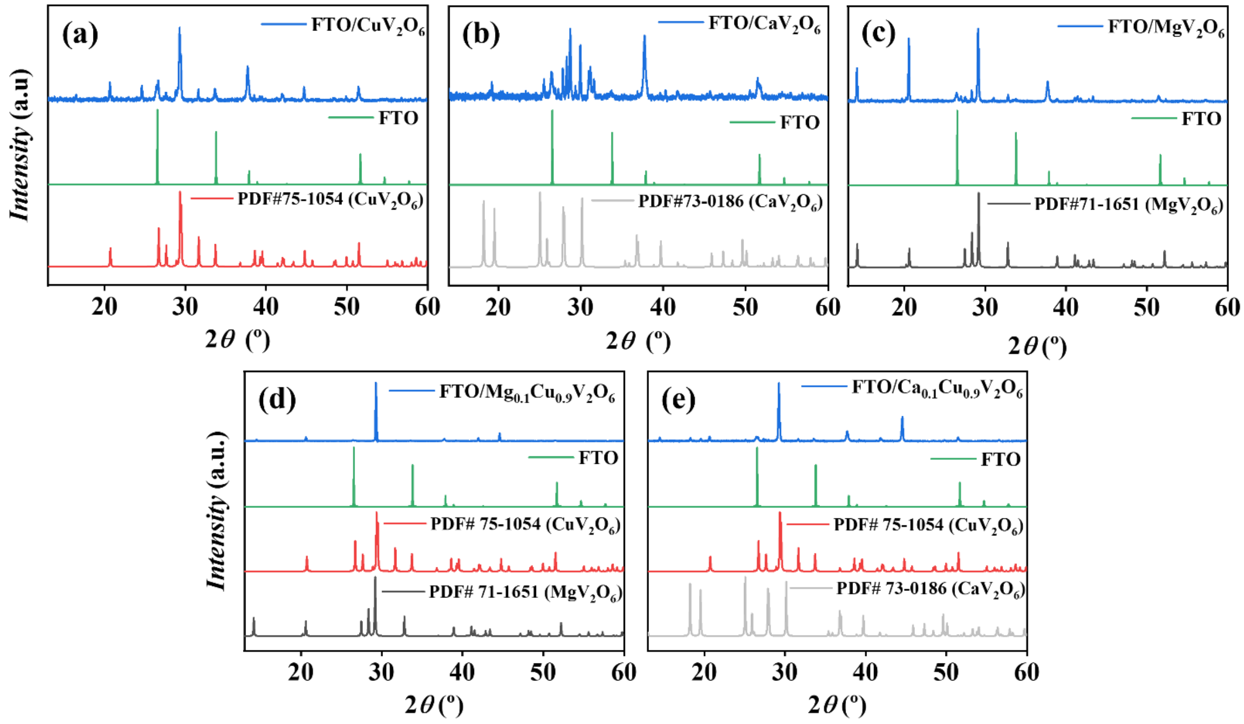

Figure S2. PXRD patterns for (a) CuV_2O_6, (b) CaV_2O_6, (c) MgV_2O_6, (d) $Mg_{0.1}Cu_{0.9}V_2O_6$, and (e) $Ca_{0.1}Cu_{0.9}V_2O_6$ and photoanodes screen-printed on FTO.

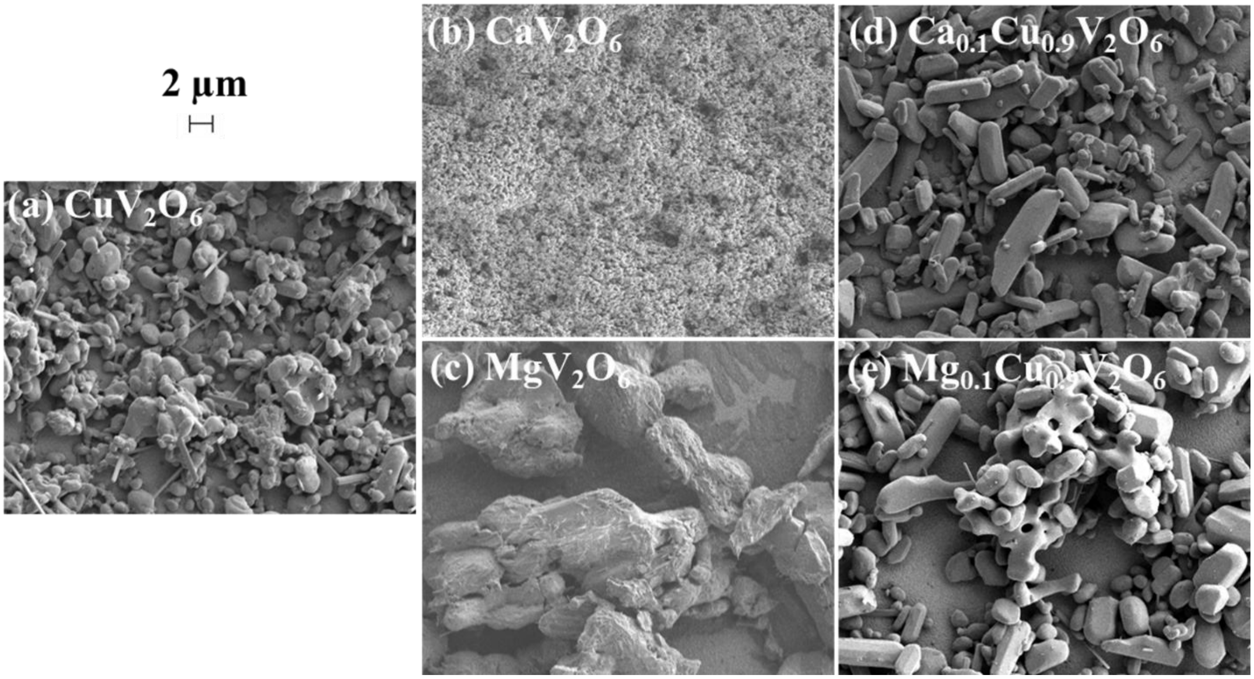

Figure S3. SEM top-view images of (a) CuV_2O_6, (b) CaV_2O_6, (c) MgV_2O_6, (d) $Ca_{0.1}Cu_{0.9}V_2O_6$, and (e) $Mg_{0.1}Cu_{0.9}V_2O_6$ electrodes screen-printed on FTO.

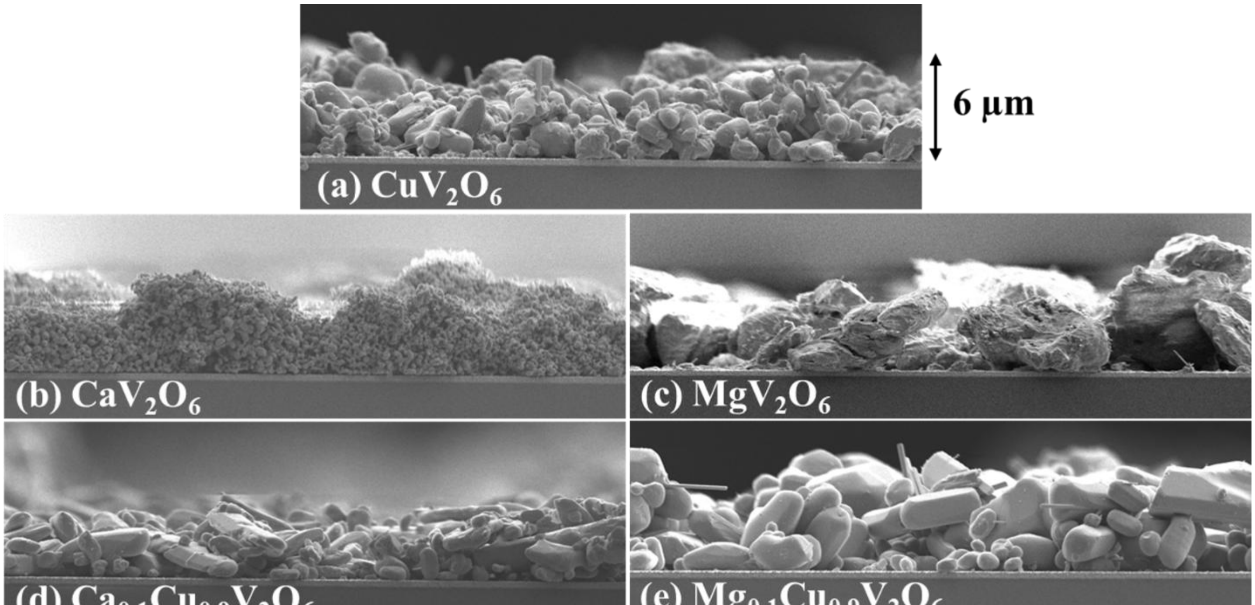

Figure S4. SEM cross-section images of (a) CuV_2O_6, (b) CaV_2O_6, (c) MgV_2O_6, (d) $Ca_{0.1}Cu_{0.9}V_2O_6$, and (e) $Mg_{0.1}Cu_{0.9}V_2O_6$ electrodes screen-printed on FTO, with 6 μm thickness.

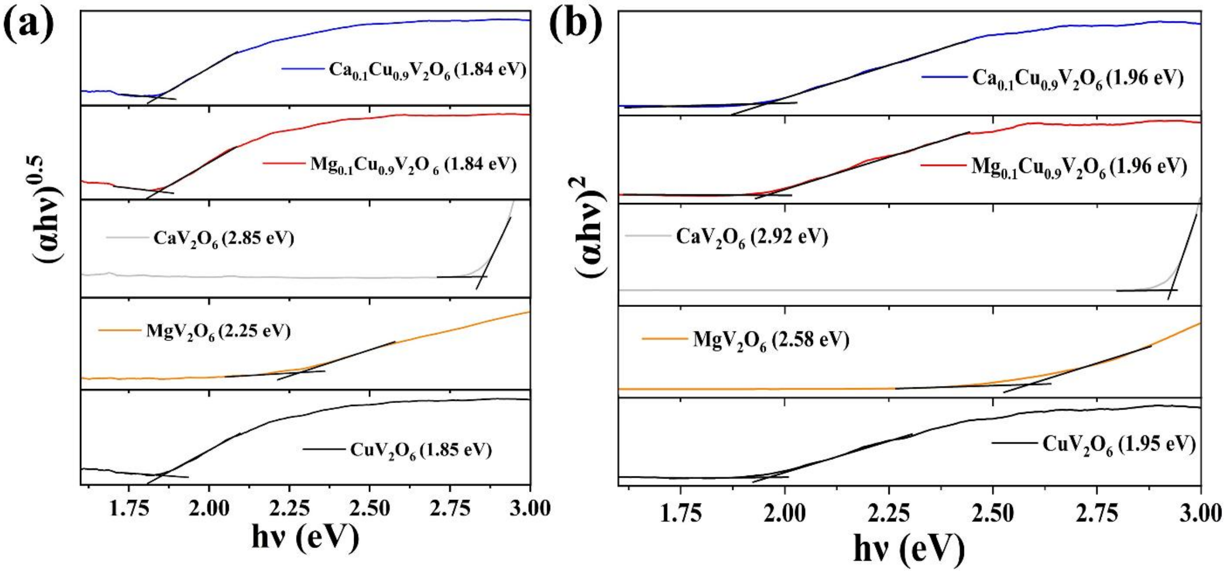

Figure S5. Kubelka-Munk analysis of the diffuse reflectance spectroscopy measurement of the films deposited onto FTO, indicating the (a) indirect and (b) direct optical band gap values for CuV_2O_6, CaV_2O_6, MgV_2O_6, $Ca_{0.1}Cu_{0.9}V_2O_6$, and $Mg_{0.1}Cu_{0.9}V_2O_6$ electrodes screen-printed on FTO.

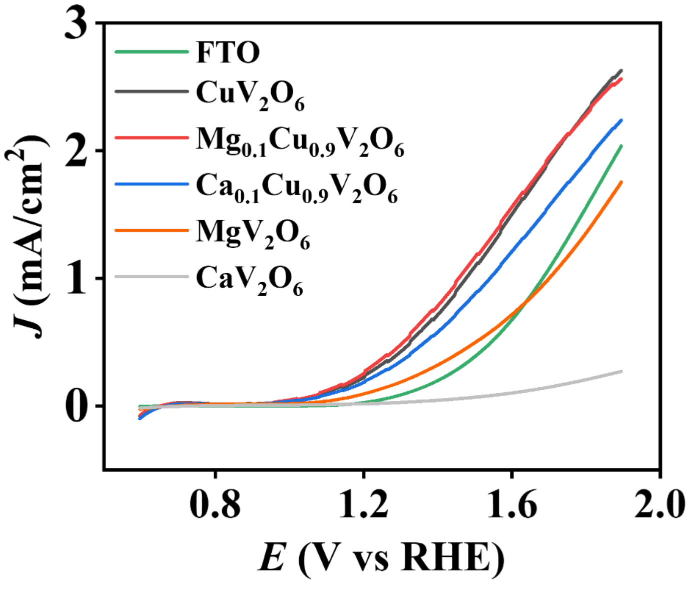

Figure S6. LSV curves with chopped light under 1 Sun illumination with AM1.5 G filter with 0.1 M phosphate buffer and 0.1 M Na_2SO_3 as hole scavenger at a scan rate of 20 mV/s for CuV_2O_6, CaV_2O_6, MgV_2O_6, $Ca_{0.1}Cu_{0.9}V_2O_6$, and $Mg_{0.1}Cu_{0.9}V_2O_6$ films screen-printed on FTO.

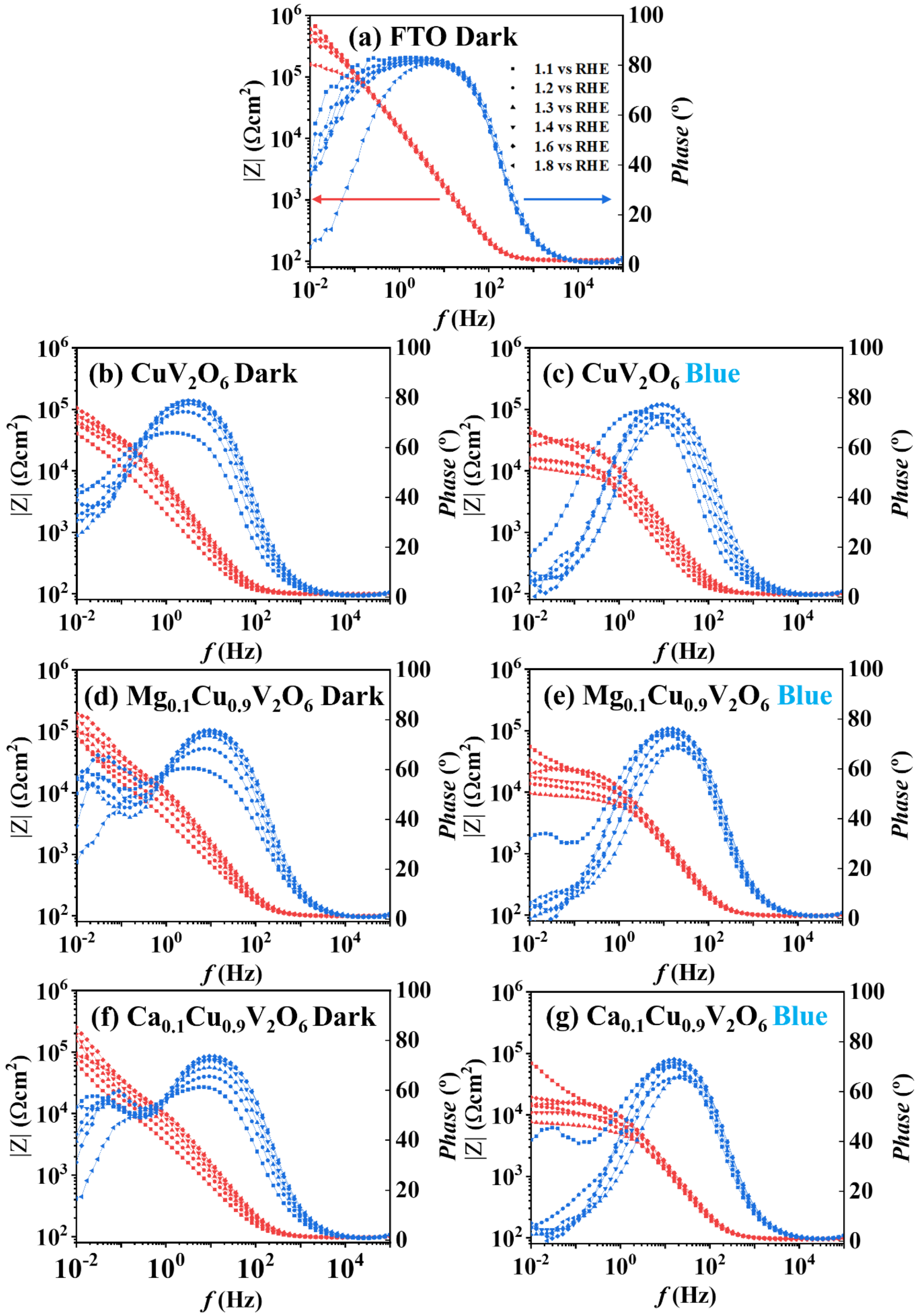

Figure S7. EIS Bode plots at different applied potentials under (a, b, c, d) dark and (e, f, g) blue (λ=455 nm) LED illumination at photon flux of 1.6 x 10^{16} $cm^{-2}s^{-1}$ in 0.1 M phosphate buffer as electrolyte for (b, c) CuV_2O_6, (d, e) $Mg_{0.1}Cu_{0.9}V_2O_6$ and (f, g) $Ca_{0.1}Cu_{0.9}V_2O_6$ photoanodes screen-printed on FTO and (a) FTO as reference.

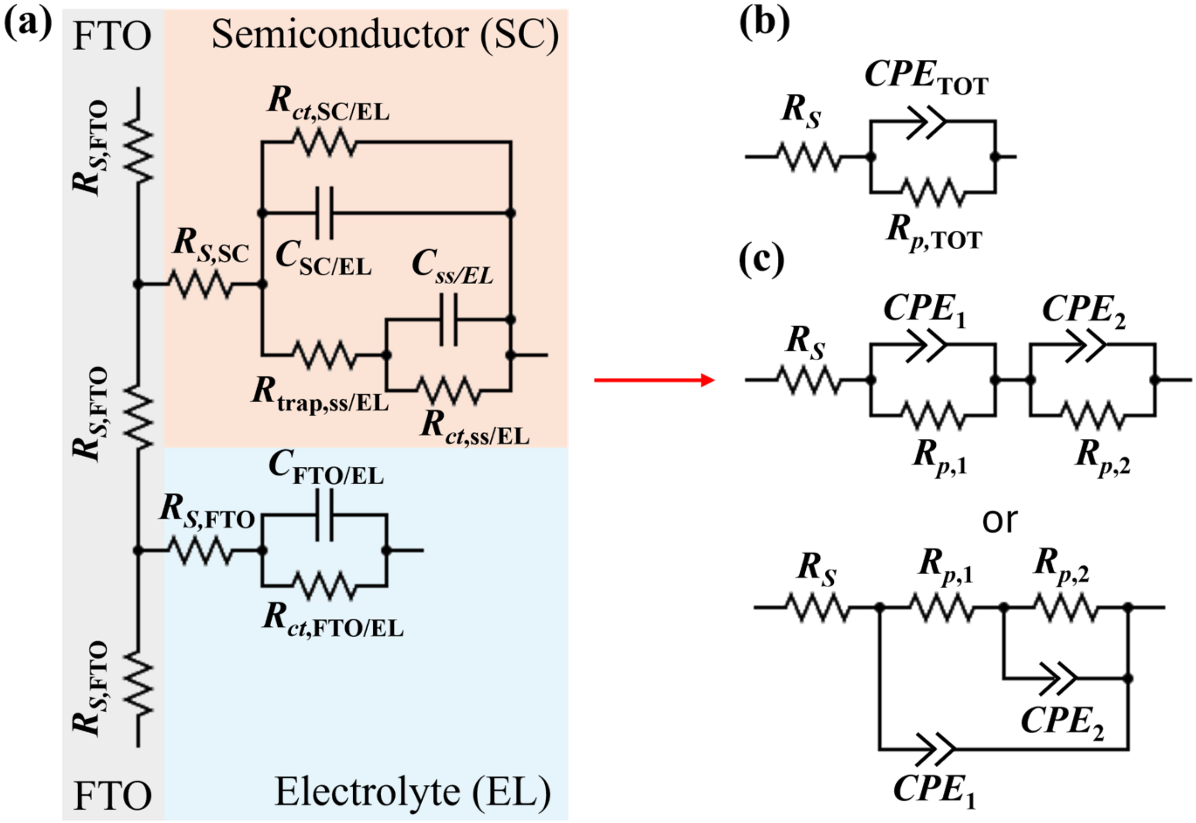

Figure S8. (a) Equivalent circuit for a typical photoelectrode with porous morphology and FTO in contact with the electrolyte solution. (b) Randles-type circuit used when only one loop is observed; and (c) simplified equivalent circuit applied when two loops are visible. R_S represents the series resistance, R_{ct} the charge transfer resistance, R_{trap} the trapping resistance, *C* the capacitance and *CPE* the constant phase element; *ss* corresponds to the surface states, SC to the semiconductor, and EL to the electrolyte solution.

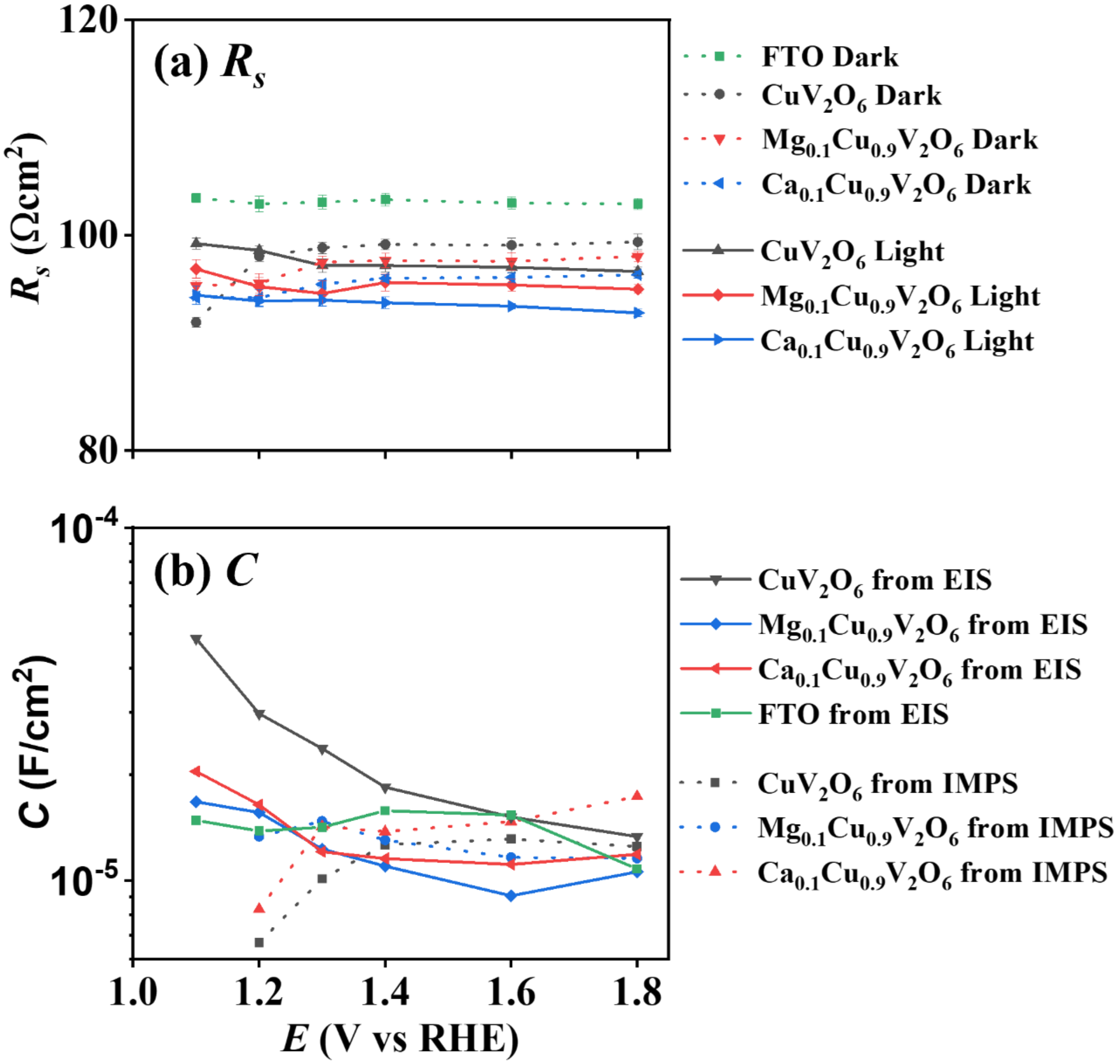

Figure S9. (a) R_S extracted from EIS; (b) C extracted from EIS and IMPS (calculated using f_{min} from IMPS and R_S from EIS) at different applied potentials using a blue (λ = 455 nm) LED at a photon flux of 1.6 x 10^{16} cm^{-2}s^{-1} in 0.1 M phosphate buffer as electrolyte for FTO, CuV_2O_6, $Mg_{0.1}Cu_{0.9}V_2O_6$, and $Ca_{0.1}Cu_{0.9}V_2O_6$ screen-printed on FTO.

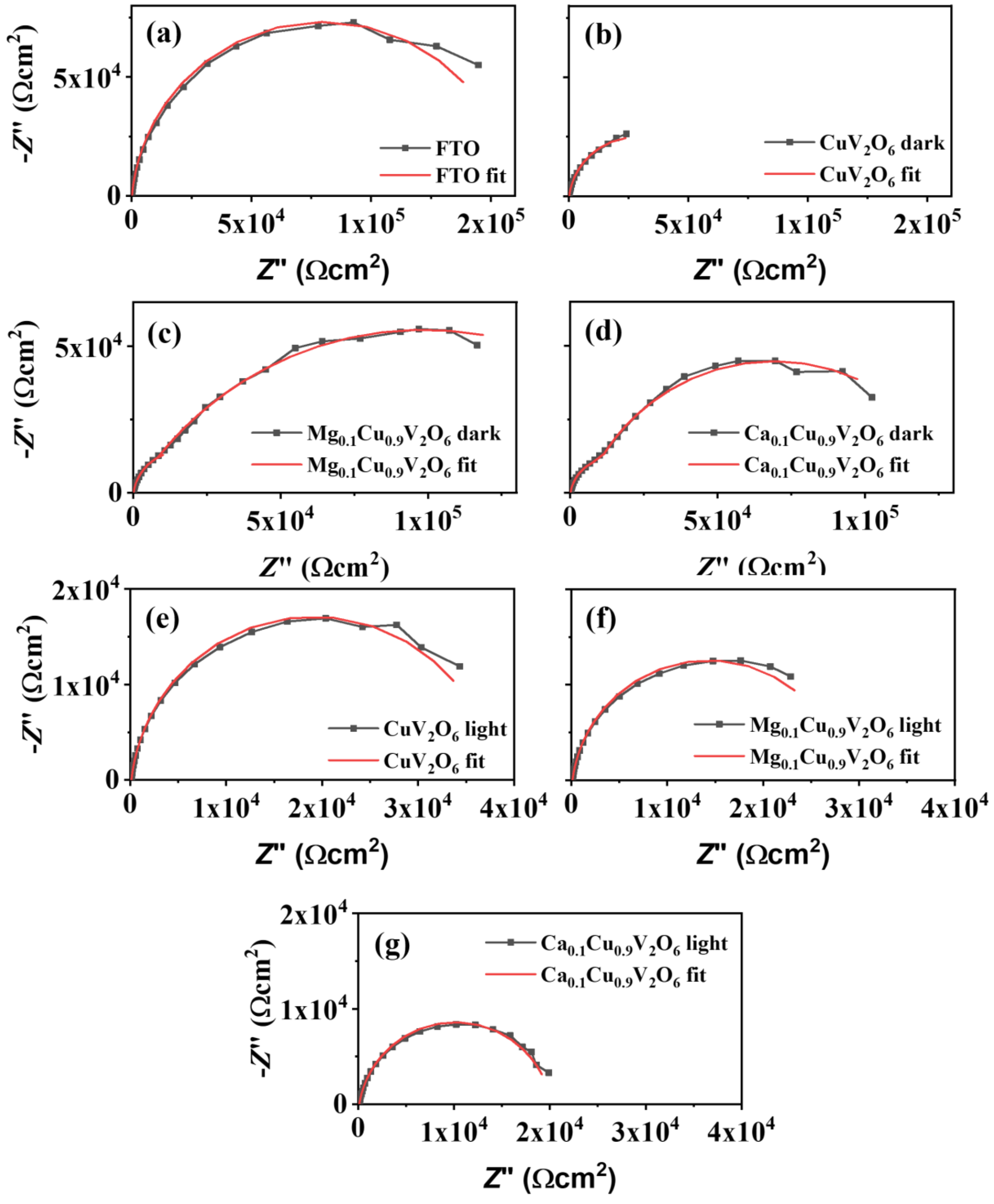

Figure S10. EIS Nyquist fitting at applied potentials of 1.8 V vs RHE in (a, b, c, d) dark and (e, f, g) blue (λ=455 nm) LED illumination at photon flux of $1.6 \cdot 10^{16}$ $cm^{-2}s^{-1}$ in 0.1 M phosphate buffer as electrolyte for (a) FTO as reference, (b, e) CuV_2O_6, (c, f) $Mg_{0.1}Cu_{0.9}V_2O_6$ and (d, g) $Ca_{0.1}Cu_{0.9}V_2O_6$ screen-printed on FTO.

Table S1. Equivalent circuit fitting parameters extracted from EIS measurements at 1.8 V vs RHE in dark and blue (λ=455 nm) LED illumination at photon flux of $1.6 \cdot 10^{16}$ $cm^{-2}s^{-1}$ in 0.1 M phosphate buffer as electrolyte.

Material	Condition at 1.8 V vs RHE	R_s	R_{tr}	*Q (CPE)*	*n*	C_{eff}
		Ωcm^2	kΩcm^2	μs^n/Ω	-	μF/cm^2
FTO	Dark	102.9 ± 0.5	125.3 ± 1.2	8.4 ± 0.1	0.95	8.6 ± 0.0
CuV_2O_6	Dark	99.4 ± 0.7	44.0 ± 1.0	22.1 ± 0.2	0.92	22.5 ± 0.1
CuV_2O_6	Blue	96.6 ± 0.4	29.8 ± 0.2	11.3 ± 0.1	0.93	10.6 ± 0.0
$Mg_{0.1}Cu_{0.9}V_2O_6$	Dark HF loop	98.0 ± 0.4	25.7 ± 0.6	11.4 ± 0.1	0.91	10.3 ± 0.0
$Mg_{0.1}Cu_{0.9}V_2O_6$	Dark LF loop	-	175.6 ± 2.4	24.7 ± 0.4	0.72	44.9 ± 0.2
$Mg_{0.1}Cu_{0.9}V_2O_6$	Blue	95.0 ± 0.4	22.3 ± 0.2	9.5 ± 0.1	0.92	8.5 ± 0.0
$Ca_{0.1}Cu_{0.9}V_2O_6$	Dark HF loop	96.3 ± 0.3	21.6 ± 0.4	12.0 ± 0.1	0.91	10.6 ± 0.0
$Ca_{0.1}Cu_{0.9}V_2O_6$	Dark LF loop	-	100.8 ± 3.4	28.5 ± 0.6	0.78	41.4 ± 0.1
$Ca_{0.1}Cu_{0.9}V_2O_6$	Blue	92.8 ± 0.3	15.8 ± 0.1	11.3 ± 0.1	0.90	9.6 ± 0.0

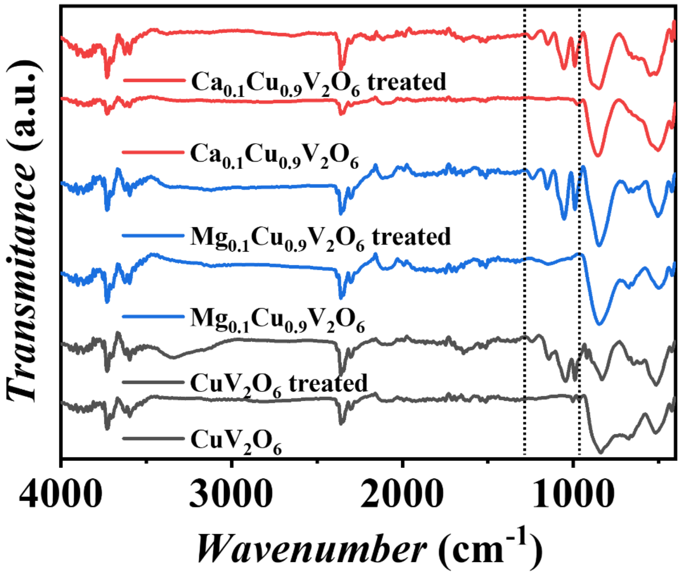

Figure S11. ATR-FTIR spectra of CuV_2O_6, $Mg_{0.1}Cu_{0.9}V_2O_6$, and $Ca_{0.1}Cu_{0.9}V_2O_6$ screen-printed on FTO, before and after photoelectrochemical measurements with phosphate buffer 0.1 M as electrolyte. The dotted lines indicate the region of interest at 1200-940 cm^{-1}.

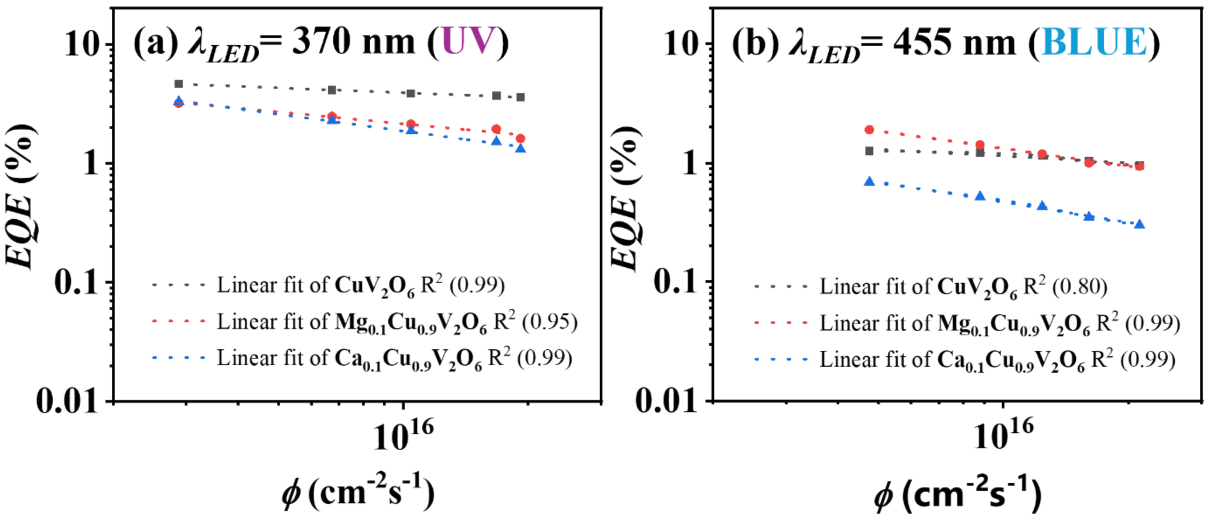

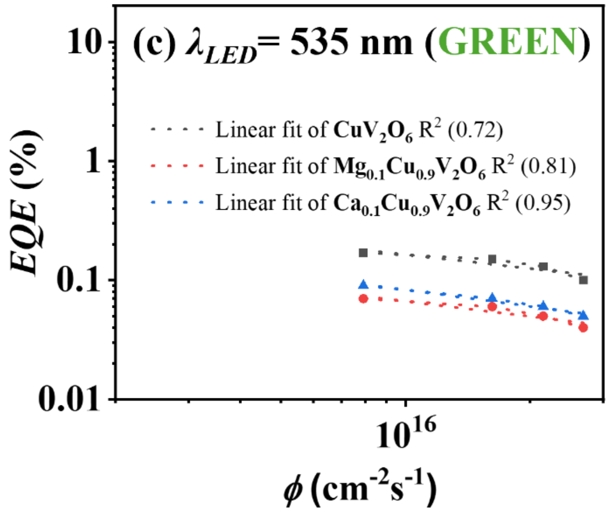

(d) slope

m	UV (370 nm)	BLUE (455 nm)	GREEN (535 nm)
CuV_2O_6	-0.13 ± 0.01	-0.19 ± 0.05	-0.39 ± 0.13
$Mg_{0.1}Cu_{0.9}V_2O_6$	-0.33 ± 0.04	-0.49 ± 0.03	-0.42 ± 0.11
$Ca_{0.1}Cu_{0.9}V_2O_6$	-0.47 ± 0.02	-0.56 ± 0.03	-0.46 ± 0.06

Figure S12. *EQE* as a function of photon flux at an applied potential of 1.8 V vs RHE with 0.1 M phosphate buffer as an electrolyte at different wavelength LED illumination (a) UV λ =370 nm, (b) blue λ =455 nm, (c) green λ =535 nm, and (d) extracted slope (*m*) from linear fit for CuV_2O_6, $Mg_{0.1}Cu_{0.9}V_2O_6$ and $Ca_{0.1}Cu_{0.9}V_2O_6$ screen-printed on FTO. The R^2 is shown for each fit.
